# Functional drug screening reveals anticonvulsants as enhancers of mTOR-independent autophagic killing of *Mycobacterium tuberculosis* through inositol depletion

**DOI:** 10.15252/emmm.201404137

**Published:** 2014-12-22

**Authors:** Mark Schiebler, Karen Brown, Krisztina Hegyi, Sandra M Newton, Maurizio Renna, Lucy Hepburn, Catherine Klapholz, Sarah Coulter, Andres Obregón-Henao, Marcela Henao Tamayo, Randall Basaraba, Beate Kampmann, Katherine M Henry, Joseph Burgon, Stephen A Renshaw, Angeleen Fleming, Robert R Kay, Karen E Anderson, Phillip T Hawkins, Diane J Ordway, David C Rubinsztein, Rodrigo Andres Floto

**Affiliations:** 1 Department of Medicine, Cambridge Institute for Medical Research, University of CambridgeCambridge, UK; 2 Cambridge Centre for Lung Infection, Papworth HospitalCambridge, UK; 3 Department of Paediatric Infectious Diseases and Allergy, Imperial College LondonLondon, UK; 4 Department of Medical Genetics, Cambridge Institute for Medical Research, University of CambridgeCambridge, UK; 5 Department of Microbiology, Immunology and Pathology, Colorado State UniversityFort Collins, Colorado, USA; 6 Department of Infection and Immunity, University of Sheffield, Western BankSheffield, UK; 7 MRC Laboratory of Molecular BiologyCambridge, UK; 8 The Inositide Laboratory, Babraham Institute, Babraham Research CampusCambridge, UK

**Keywords:** autophagy, multidrug-resistant, myo-inositol, tuberculosis

## Abstract

*Mycobacterium tuberculosis* (MTB) remains a major challenge to global health made worse by the spread of multidrug resistance. We therefore examined whether stimulating intracellular killing of mycobacteria through pharmacological enhancement of macroautophagy might provide a novel therapeutic strategy. Despite the resistance of MTB to killing by basal autophagy, cell-based screening of FDA-approved drugs revealed two anticonvulsants, carbamazepine and valproic acid, that were able to stimulate autophagic killing of intracellular *M. tuberculosis* within primary human macrophages at concentrations achievable in humans. Using a zebrafish model, we show that carbamazepine can stimulate autophagy *in vivo* and enhance clearance of *M. marinum*, while in mice infected with a highly virulent multidrug-resistant MTB strain, carbamazepine treatment reduced bacterial burden, improved lung pathology and stimulated adaptive immunity. We show that carbamazepine induces antimicrobial autophagy through a novel, evolutionarily conserved, mTOR-independent pathway controlled by cellular depletion of myo-inositol. While strain-specific differences in susceptibility to *in vivo* carbamazepine treatment may exist, autophagy enhancement by repurposed drugs provides an easily implementable potential therapy for the treatment of multidrug-resistant mycobacterial infection.

See also: AJ Olive & CM Sassetti (February 2015)

## Introduction

The increase in infections with multidrug-resistant (MDR) and extensive drug-resistant (XDR) *Mycobacterium tuberculosis* (MTB), requiring longer and more toxic drug regimens which often fail, has created significant barriers to effective treatment of tuberculosis in both well-resourced and resource-poor settings (Nathanson *et al*, 2010; Lienhardt *et al*, 2012) and illustrates the pressing need for new, more effective therapies. Although some progress has been made in developing novel antibiotics (www.newtbdrugs.org/pipeline.php), we have pursued an alternative therapeutic strategy of trying to stimulate intracellular killing of MTB within macrophages through pharmacological induction of autophagy.

Autophagy is a fundamental, evolutionarily conserved mechanism used by cells to degrade cytoplasmic protein aggregates and organelles (Mizushima *et al*, 2008) but can also target intracellular bacteria, including *M. tuberculosis*, for lysosomal degradation (Gutierrez *et al*, 2004; Deretic & Levine, 2009). Greater understanding of how *M. tuberculosis* triggers basal autophagy has recently emerged, suggesting that ESAT6-dependent phagosome rupture permits ubiquitin tagging of mycobacteria, LC3 recruitment through NDP52 and p62 adaptor binding and engagement of TBK1 (Watson *et al*, 2012), an enzyme critical for inflammasome activation (Pilli *et al*, 2012). However, the mechanisms permitting intracellular mycobacteria to resist basal autophagic killing and persist within macrophages remain poorly understood.

Intracellular killing of *M. tuberculosis* can be enhanced through mTOR-dependent stimulations of autophagy (Gutierrez *et al*, 2004). While effective *in vitro*, this approach is probably unsuitable for clinical use, since mTOR inhibition also leads to immunosuppression (McMahon *et al*, 2011). We therefore wondered whether there might be existing clinical drugs that could stimulate mTOR-independent autophagic killing of *M. tuberculosis*, which could be rapidly repurposed as novel treatments for multidrug-resistant mycobacteria.

Here, we show that screening of FDA-approved drugs identified compounds able to stimulate autophagic killing of mycobacteria at therapeutic concentrations. One such compound, carbamazepine, triggers autophagy independently of mTOR (revealing a novel myo-inositol-dependent activation pathway) and effectively targets multidrug-resistant *M. tuberculosis in vivo* stimulating both innate and adaptive immunity.

## Results

### FDA-approved drugs able to stimulate autophagic killing of mycobacteria

**Figure 1:**
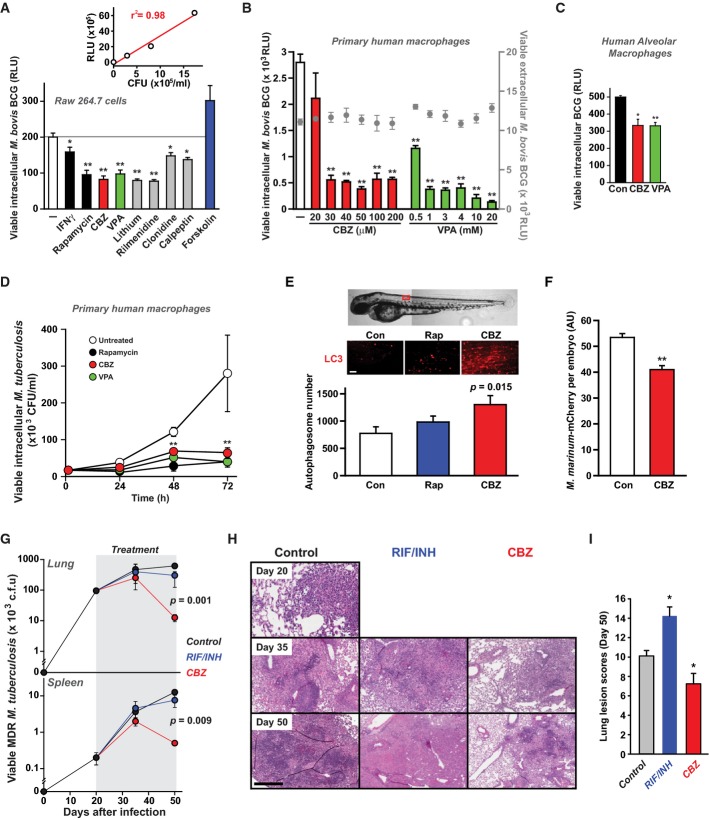
Anticonvulsants stimulate killing of mycobacteria *in vitro* Screening drugs for effects on survival of intracellular mycobacteria in macrophages. RAW 264.7 cells were infected with a luminescent strain of *M. bovis* BCG (BCG-*lux*) for 1 h, washed and treated for 24 h with vehicle alone (*white*), known autophagy enhancers (interferon-γ (IFNγ), 200 ng/ml and rapamycin, 200 nM; *black*), known mTOR-independent autophagy inhibitor (forskolin; 24 μM; *blue*), carbamazepine (CBZ, 50 μM; *red*), valproic acid (VPA, 3 mM; *green*) and other examples of hits from a large screen of compounds enhancing intracellular killing of mycobacteria (lithium, 10 mM; rilmenidine, 1 μM; clonidine, 1 μM; calpeptin, 50 μM; *grey*). *P*-values, unpaired Student's *t*-test (*n *≥* *6) (compared to vehicle alone): IFNγ 0.03; rapamycin 0.003, CBZ 0.001, VPA 0.001, lithium 0.001, rilmenidine 0.001; clonidine 0.02; calpeptin 0.01. *Inset:* Correlation between measurements of colony-forming units (CFU) and luminescence (RLU) for cultures of *M. bovis* BCG-*lux* as previously described (Kampmann *et al*, 2000).

We have previously shown that novel mTOR-independent pathways exist to activate autophagy (Sarkar *et al*, 2005; Williams *et al*, 2008). Through high-throughput screening of a library of 214 compounds enriched for drugs already FDA-approved for non-infectious indications (see Methods), we identified several compounds able to activate mTOR-independent autophagic killing of intracellular mycobacteria (after 24-h treatment). We focused on two anticonvulsant drugs, carbamazepine (CBZ) and valproic acid (VPA) which stimulated killing of mycobacteria (*M. bovis* BCG) within primary human monocyte-derived and alveolar macrophages at concentrations achievable during therapeutic dosing, while not affecting mycobacterial survival in the absence of cells or macrophage viability (Fig1A–C; Supplementary Fig S1 & Supplementary Table 1). We also found that both CBZ and VPA stimulated intracellular killing of *M. tuberculosis* (H37Rv) within primary human macrophages (Fig1D). Since the lowest effective concentration of VPA was at the upper limit of what might be safely achieved in humans, we focused on CBZ for further therapeutic evaluation *in vivo*. In zebrafish expressing fluorescent ATG8, treatment with CBZ for 24 h (in the presence of low-dose chloroquine to delay degradation of autophagosomes) led to a significantly greater accumulation of autophagosomes than in fish treated with vehicle alone or rapamycin (Fig1E). Furthermore, CBZ was also able to enhance clearance of *M. marinum* from infected zebrafish embryos (Fig1F), an established model of mycobacterial infection (Lesley & Ramakrishnan, 2008). We then determined the impact of CBZ *in vivo* in a mouse model of *M. tuberculosis* infection. Since conventional antibiotic resistance should not influence autophagic killing of mycobacteria, we examined the effect of CBZ in mice infected via aerosol with a highly virulent multidrug-resistant *M. tuberculosis* (MDR-TB) strain (CSU 87; Park *et al*, 2006). Treatment with CBZ (at a dose mirroring therapeutic administration in humans) significantly reduced MDR-TB burden in lungs and spleen, diminished inflammatory pulmonary infiltrates and decreased lung lesion scores, whereas treatment with first line anti-mycobacterial antibiotics rifampicin and isoniazid (RIF/INH), as expected, had no effect (Fig1G–I). However, in further studies on mice infected with a less virulent MDR-TB strain (CSU39; Rhoades & Orme, 1997), or a drug-sensitive isolate (SA310; Palanisamy *et al*, 2009), CBZ treatment had little or no effect *in vivo* (Supplementary Fig S2).

Effects on intracellular survival of *M. bovis* BCG-*lux* of treatment with varying concentrations of CBZ (*red*) or VPA (*green*). *P*-values, unpaired Student's *t*-test (*n = *6) (compared to vehicle alone) for CBZ 30 μM: 9 × 10^−7^, 40 μM: 1.9 × 10^−9^, 50 μM: 1.3 × 10^−9^, 100 μM: 1.6 × 10^−8^, 200 μM: 2.7 × 10^−9^, VPA 0.5 mM: 6.9 × 10^−8^, 1 mM: 1.6 × 10^−9^, 3 mM: 4.3 × 10^−10^, 4 mM: 4.2 × 10^−9^, 10 mM: 1.1 × 10^−9^, 20 mM: 4.2 × 10^−10^. These compounds had no effect on cell-free mycobacterial viability (*grey circles*).

Anticonvulsants enhance intracellular killing of mycobacteria within human alveolar macrophages. Alveolar macrophages, obtained from the broncho-alveolar lavage fluid of three individuals, were infected with *M. bovis* BCG-*lux* and then treated with carbamazepine (CBZ, 50 μM; *red*), valproic acid (VPA, 3 mM; *green*) or vehicle alone (Con; *black*). Viable intracellular mycobacteria were determined after 24 h of treatment. Unpaired Student's *t*-test (*n = *3) (compared to vehicle alone): CBZ 0.011; VPA 0.0009.

Enhanced killing of intracellular *M. tuberculosis* (H37Rv) within primary human macrophages by treatment with CBZ (50 μM; *red*), VPA (3 mM; *green*) and rapamycin (200 nM; *black*), compared to control (vehicle alone; *white*). Unpaired Student's *t*-test (*n = *6) (compared to vehicle alone): rapamycin 48 h 0.00002, rapamycin 72 h 0.00005, CBZ 48 h 0.00021, CBZ 72 h 0.00022, VPA 48 h 0.0002, VPA 72 h 0.00049.

*In vivo* induction of autophagy by CBZ (50 μM; *red*) and rapamycin (RAP; 1 μM; *blue*) compared to vehicle control (*white*) monitored in zebrafish expressing fluorescent ATG8 co-treated with chloroquine to delay degradation of autophagosomes.

Wild-type zebrafish were injected with a red fluorescently tagged *M. marinum* strain M into the yolk sac circulation valley at 28 hpf. Larvae were imaged at 120 hpf by confocal microscopy and the total mycobacteria-associated fluorescence quantified using Volocity® software. Data expressed as mean ± SEM (*n* ≥ 13 fish performed as 3 independent experiments). *P*-values, unpaired Student's *t*-test (compared to vehicle alone): 0.0035.

Mice infected via aerosol with a highly virulent clinical strain of multidrug-resistant *M. tuberculosis* CSU 87 were treated from day 20 post-infection with carbamazepine (CBZ, 50 μg/kg *i.p*. daily), rifampicin/isoniazid (RIF/INH) or vehicle control (*n *=* *5 per time point per group). CBZ treatment for 30 days resulted in (G) significantly less viable bacteria detected in lung and spleen, (H) reduced inflammatory pulmonary infiltrates compared to RIF/INH-treated or control animals and (I) decreased lung lesion scores. Unpaired Student's *t*-test (*n = *5) (compared to vehicle alone): RIF/INH 0.007; CBZ 0.037. Data information: *P < 0.05; **P < 0.005.

### Carbamazepine promotes innate and adaptive immunity during mycobacterial infection

**Figure 2:**
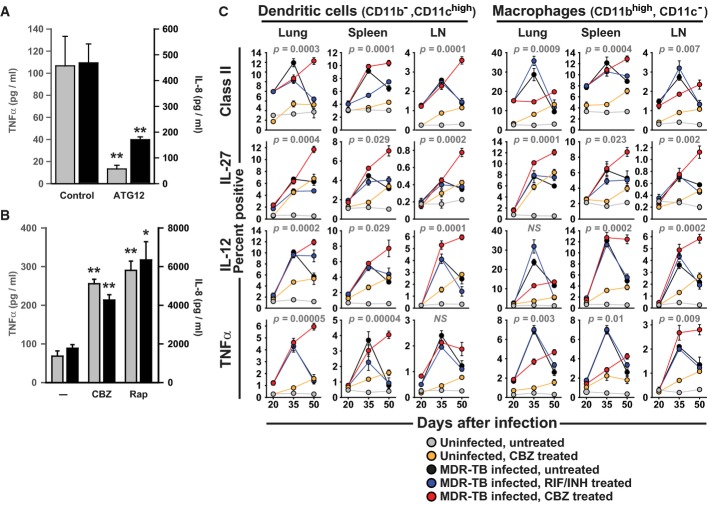
Carbamazepine promotes innate and adaptive immunity during mycobacterial infection Primary human macrophages infected with *M. bovis* BCG were treated with carbamazepine (CBZ, 50 μM) or rapamycin (Rap; 200 nM). IL-8 (*black*) and TNFα (*grey*) were measured in supernatants collected 24 h after infection.

We found that macrophages infected with mycobacteria demonstrated autophagy-dependent pro-inflammatory cytokine production (Fig2A) and *in vitro* treatment with CBZ, as well as rapamycin, increased TNFα and IL-8 release (Fig2B), potentially through enhanced degradative generation of ligands for intracellular pattern recognition receptors. We also examined the effect of CBZ on cytokine production and other components of host immunity to mycobacteria *in vivo*. Treatment of infected mice with CBZ resulted in significantly greater TNFα, IL-12 and IL-27 production and MHC class II surface expression by dendritic cells and macrophages (Fig2C), which are all associated with favourable outcomes clinically and suggest that CBZ, in addition to enhancing intracellular killing, might also promote adaptive immune responses. Supporting evidence comes from analysis of lung CD4^+^ T cells tracking cytokine production as well the kinetics of effector (CD4^+^CD44^hi^CD62^lo^), and memory (CD4^+^CD44^hi^CD62L^hi^) T cells influx. CBZ treatment increases the numbers of central memory and IFNγ-secreting T cells while reducing FoxP3^+^ IL-10^+^ regulatory T cells (Supplementary Fig S3). These findings are in keeping with previous reports suggesting that autophagy stimulation may improve antigenic peptide production during mycobacterial infection (Jagannath *et al*, 2009). We also noticed a mild pro-inflammatory effect of CBZ treatment in uninfected animals (Fig.2C), potentially reflecting non-specific immune stimulation.

Levels of IL-8 (*black*) and TNFα (*grey*) released by *M. bovis* BCG-infected primary human macrophages were autophagy dependent, measured following siRNA knock-down of the critical autophagy protein, ATG12, or control siRNA (control). *P*-values, unpaired Student's *t*-test (*n = *3) ATG12 siRNA vs siControl: TNFα 0.0006; IL-8 0.0012.

Carbamazepine increases pro-inflammatory cytokine secretion by mycobacteria-infected human macrophages. *P*-values, unpaired Student's *t*-test (*n = *5) (compared to vehicle alone): CBZ TNFα 0.00048, IL-8 0.0017; Rap TNFα 0.001, IL-8 0.0089.

Analysis (by flow cytometry) of intracellular cytokines (IL-27, IL-12, TNFα) and MHC class II surface expression for dendritic cells (CD11c^+^) and macrophages (CD11b^+^) from lung, spleen and draining lymph nodes of mice that were uninfected and untreated (*grey*), uninfected and CBZ-treated (*orange*), infected with multidrug-resistant *M. tuberculosis* (MDR-TB) and untreated (*black*), MDR-TB infected and treated with rifampicin and isoniazid (RIF/INH; *blue*) or MDR-TB infected and treated with carbamazepine (CBZ, *red*).

Data Information: * *P *< 0.05; ** *P *< 0.005. All experiments were carried out at least in triplicate and on at least 2 separate occasions.

### Carbamazepine triggers mTOR-independent autophagy through depletion of cellular myo-inositol

**Figure 3:**
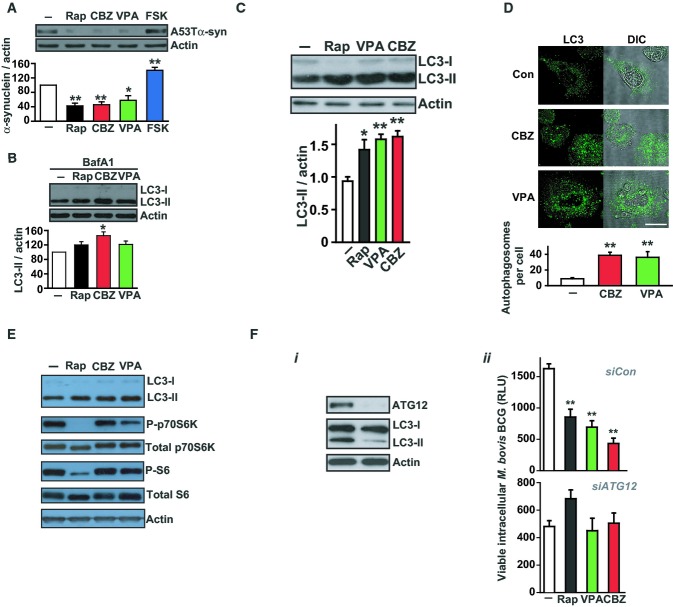
Anticonvulsants stimulate autophagosome formation *in vitro* The anticonvulsants carbamazepine and valproic acid enhance autophagic clearance of cytosolic substrate. We monitored the clearance of the known autophagy substrate (A53T) α-synuclein in stable inducible PC12 cells by Western blot. The A53T α-synuclein transgene was induced with doxycycline for 48 h and then switched off (by antibiotic removal) before cells were treated with carbamazepine (CBZ, 50 μM; *red*), valproic acid (VPA, 3 mM; *green*), rapamycin (Rap, 200 nM; *black*), forskolin (FSK, 10 μM; *blue*) or vehicle alone (DMSO; *white*) for a further 24 h. *P*-values, paired Student's *t*-test (*n = *3) (compared to vehicle alone): Rap 0.0011; CBZ 0.0017; VPA 0.02; FSK 0.005.

We then examined the molecular mechanisms of action of CBZ and VPA. Both compounds enhanced autophagosome synthesis and clearance of known autophagic substrates in cell lines (Fig3A and B) and increased autophagosome numbers in human macrophages, assessed by LC3 Western blot and confocal microscopy (Fig3C and D). We confirmed that CBZ and VPA trigger autophagosome formation independently of mTOR (Fig3E). When autophagy was blocked by siRNA knock-down of ATG12, CBZ and VPA failed to stimulate clearance of intracellular mycobacteria (*M. bovis* BCG), validating that their effects were mediated through enhanced autophagic killing (Fig3Fi, ii).

Anticonvulsants enhance autophagosome synthesis in primary human macrophages. In the presence of a saturating concentration of bafilomycin A_1_ (400 nM) which blocks autophagosome–lysosome fusion, levels of LC3-II reflect autophagosome production and were quantified in human macrophages (from a different healthy individual in 3 separate experiments) treated with rapamycin (Rap, 200 nM; *black*), carbamazepine (CBZ, 50 μM; *red*), valproic acid (VPA, 3 mM; *green*) or vehicle alone (DMSO; *white*). *P*-values, paired Student's *t*-test (*n = *3) (compared to vehicle alone): CBZ 0.026.

Increases in LC3-II levels (assessed by Western blot) in primary human macrophages infected with *M. bovis* BCG after 4 h treatment with rapamycin (Rap; 200 nM), valproic acid (VPA, 3 mM) and carbamazepine (CBZ, 50 μM) compared to controls. *P*-values, paired Student's *t*-test (*n = *3) (compared to vehicle alone): Rap 0.02; VPA 0.004; CBZ 0.003.

Treatment of *M. bovis* BCG-infected human macrophages with CBZ (50 μM) or VPA (3 mM) increased autophagosome number, assessed by confocal microscopy of cells stained with LC3-specific antibody (*green*) with quantification of the number of autophagosomes (defined as LC3 +  vesicles ≥ 1 μm diameter) per cell shown below. *P*-values, unpaired Student's *t*-test (*n = *3) (compared to vehicle alone): CBZ 0.001, VPA 0.003. Scale bar represents 10 μm.

In contrast to rapamycin (Rap), carbamazepine (CBZ) treatment of macrophages, while increasing LC3-II levels, does not alter mTOR-dependent signalling (monitored by changes in phosphorylation of S6 and p70S6 kinase).

Enhanced intracellular killing of mycobacteria by anticonvulsants is mediated through autophagy. SiRNA knock-down of ATG12 in primary human macrophages blocks autophagy leading to (i) reduced LC3-II levels and (ii) loss of CBZ- (50 μM) and VPA- (3 mM) induced enhancement of intracellular killing of *M. bovis* BCG. *P*-values, unpaired Student's *t*-test (*n = *3) (compared to vehicle alone): Rap 0.002; VPA 0.001; CBZ 0.0001. Data information: * *P *< 0.05; ** *P *< 0.005. All experiments were carried out at least in triplicate and on at least 3 separate occasions.

**Figure 4:**
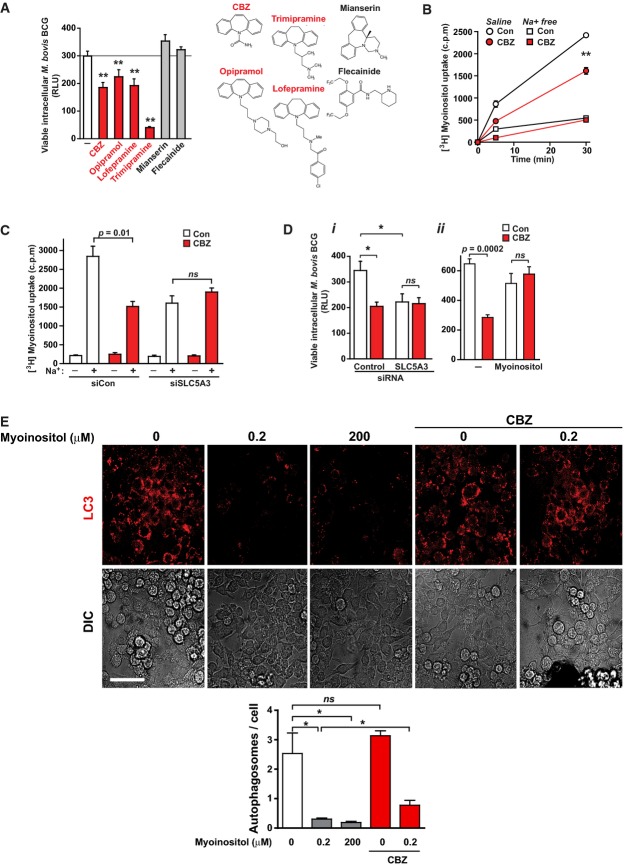
Carbamazepine triggers mTOR-independent autophagy through myo-inositol depletion Effect on intracellular killing of luminescent mycobacteria in primary human macrophages of CBZ (50 μM), other dibenzazepines (opipramol, 100 ng/ml; lofepramine, 200 ng/ml; trimipramine, 50 ng/ml), mianserin (200 ng/ml) and the cardiac-specific SCN5A antagonist, flecainide (2 μg/ml). *P*-values, unpaired Student's *t*-test (*n* = ≥ 6) (compared to vehicle alone): CBZ 0.0001; opipramol 0.0005; lofepramine 0.0001; trimipramine 1 × 10^−5^.

We next focused on the mechanism by which CBZ induced autophagy in human macrophages. We first looked for the presence of known molecular targets of CBZ in primary human macrophages and identified the voltage- and stretch-activated sodium (Na^+^) channel SCN5A (Supplementary Fig S4; Carrithers *et al*, 2011), but not other SCN channels. We observed that SCN5A-blocking dibenzazepines (Lipkind & Fozzard, 2010), but not the cardiac-specific inhibitor flecainide, also enhanced killing of intracellular *M. bovis* BCG (Fig4A), with no adverse cytotoxic effects (Supplementary Fig S5), suggesting that SCN5A might be the molecular target of CBZ on macrophages. Since SCN5A may regulate the sodium–inositol co-transporter, SLC5A3, in neuronal cell lines (as suggested previously; Yamashita *et al*, 1999), we tested whether CBZ could induce autophagic clearance of mycobacteria through inhibition of myo-inositol uptake. Consistent with this model, CBZ blocked Na^+^-dependent myo-inositol uptake in macrophages (Fig4B); siRNA knock-down of SLC5A3 led to reduced Na^+^-dependent myo-inositol uptake, increased autophagosome formation (Supplementary Fig S6) and enhanced intracellular killing of *M. bovis* BCG with no additional effect of CBZ treatment (Fig4C and D). Furthermore, co-incubation of macrophages with high concentrations of myo-inositol blocked the activity of CBZ to enhance intracellular killing of mycobacteria (Fig4D) and induce autophagy (Supplementary Fig S7). To confirm that myo-inositol depletion was sufficient to activate autophagy, we incubated cells in cell culture media with varying amounts of myo-inositol and observed stimulation of autophagosome formation, by both confocal microscopy and Western blot, and enhanced clearance of autophagic protein substrates (Fig4E, Supplementary Fig S7).

CBZ inhibits Na^+^-dependent myo-inositol uptake by macrophages. Rates of [^3^H]myo-inositol accumulation within cells were determined in the presence (*squares*) or absence (*circles*) of extracellular Na^+^ during treatment with CBZ (50 μM; *red*) or vehicle alone (*white*). *P*-values, unpaired Student's *t*-test (*n = *6) (compared to vehicle alone): CBZ 9.2 × 10^−8^.

Sodium-dependent myo-inositol uptake (at 5 min) by macrophages is reduced by siRNA knock-down of the Na^+^–inositol co-transporter SLC5A3 (SMIT-1) under which conditions co-treatment with CBZ (50 μM; *red*) caused no additional inhibition of inositol uptake.

Effect on intracellular killing of luminescent mycobacteria in primary human macrophages of (i) siRNA knock-down of the Na^+^–inositol co-transporter SLC5A3 (SMIT-1) (*P*-values, unpaired Student's *t*-test (*n = *3) siSLC5A3 compared to siControl: 0.009; siControl + CBZ compared to vehicle alone: 0.0067) and (ii) incubation with excess extracellular myo-inositol with (*red*) or without (*white*) treatment with CBZ at 50 μM (*n = *3).

Effect of depleting and adding excess myo-inositol on LC3-II mCherry autophagosome formation in RAW 264.7 macrophages. Myo-inositol depletion increases autophagosome formation (*white*), with no significant additional change with CBZ treatment (*red*). Excess myo-inositol further reduces autophagosome number (*grey*). *P*-values, unpaired Student's *t*-test (*n = *3) (compared to myo-inositol-free): 0.2 μM 0.013, 200 μM 0.01; 0.2 μM compared 0.2 μM + CBZ: 0.023. Scale bar represents 40 μm. Data information: * *P *< 0.05; ** *P *< 0.005. All experiments were carried out at least in triplicate and on at least 3 separate occasions.

### Carbamazepine stimulates autophagy through IP_3_ depletion and AMPK activation

**Figure 5:**
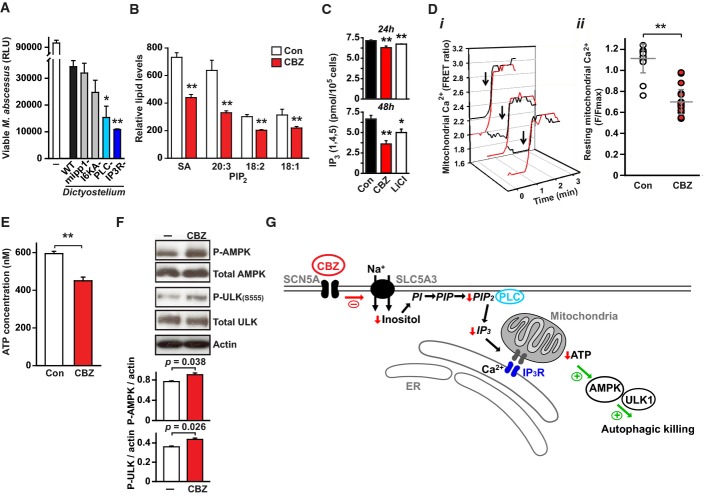
Carbamazepine induces autophagy through decreased IP_3_ signalling Phospholipase C and IP_3_ receptor regulate autophagic killing of mycobacteria in *Dictyostelium*. Wild-type (WT, *black*) and mutant *Dictyostelium* AX2 strains were grown axenically and incubated with *M. abscessus-lux* (Renna *et al*, 2011) at an MOI of 0.01:1. *M. abscessus-lux* with no *Dictyostelium* (*white*) were grown under identical conditions as a control. Viable mycobacteria were quantified by measuring luminescence at 2 h post-infection. Mutant strains tested: *mipp1*− (multiple inositol polyphosphate phosphatase null), I6KA− (inositol hexakisphosphate phosphate kinase null); plc− (phospholipase C null); IP3R− (inositol (1,4,5)-trisphosphate receptor null mutant iplA-). *P*-values, unpaired Student's *t*-test (*n = *3) (compared to wild-type): PLC− 0.01; IP3R− 0.007.

To further define which signalling components might mediate inositol-regulated intracellular killing of mycobacteria, we turned to *Dictyostelium,* which provides a robust model for examining both intracellular killing of mycobacteria (Hagedorn *et al*, 2009) and inositol signalling (Williams *et al*, 1999). We first confirmed that killing of *M. abscessus* by *Dictyostelium* was impaired by autophagy inhibitors (Supplementary Fig S8). Using published deletion mutants (Traynor *et al*, 2000; Luo *et al*, 2003; Kortholt *et al*, 2007; Cho *et al*, 2008), we observed enhanced killing of *M. abscessus* by strains deficient in phospholipase c (PLC−) or inositol (1,4,5)-trisphosphate receptor (IP3R−; Fig5A), suggesting a critical role for IP_3_ in regulation of autophagic killing. As anticipated following CBZ-induced depletion of cellular myo-inositol, we also observed reductions in basal levels of phosphatidylinositol bisphosphate (PIP_2_; Fig5B) and inositol (1,4,5)-trisphosphate (IP_3_; Fig5C) in macrophages. In agreement with the recently proposed role of basal IP_3_ in regulating mitochondrial function and thereby autophagy (Cárdenas *et al*, 2010), CBZ treatment in macrophages reduced levels of mitochondrial calcium (Fig5D), decreased cellular ATP concentrations (Fig.5E) and consequently increased phosphorylation of AMP kinase and ULK1 (Fig5F, Supplementary Fig S9), which are established triggers of mTOR-independent autophagy (Cárdenas *et al*, 2010; Alers *et al*, 2011; Egan *et al*, 2011). These data are consistent with a model for inositol regulation of autophagic killing in macrophages which could potentially be amenable to therapeutic intervention at multiple steps. Thus, depletion of cytosolic myo-inositol leads to reduced basal IP_3_ (through decreased available PIP_2_) which, by reducing mitochondrial Ca^2+^ entry, results in decreased ATP production ultimately activating AMP kinase/ULK1-dependent stimulation of autophagy (Fig5G).

Quantification by mass spectroscopy of levels of C18:0 PIP_2_ species in macrophages following treatment with CBZ (*red*) or vehicle control (*white*). SA- C18:0/C20:4. *P*-values, unpaired Student's *t*-test (*n = *3) (compared to vehicle alone): SA 6.6 × 10^−9^, 20:3 1.6 × 10^−6^, 18:2 3.5 × 10^−8^, 18:1 3.2 × 10^−4^.

Levels of basal IP_3_ (1,4,5) in macrophages following treatment with CBZ (*red*), lithium (*white*) or vehicle alone (*black*) for 24 h (*top*) or 48 h (*bottom*). *P*-values, unpaired Student's *t*-test (*n *≥* *3) (compared to vehicle alone): CBZ 24 h 0.00007; CBZ 24 h 0.0013; LiCl 24 h 0.00005; LiCl 48 h 0.04.

Resting levels of mitochondrial calcium (monitored by *mt-Cameleon* sensor expression) are reduced by CBZ treatment (*red*) compared to controls. (i) Representative calcium recordings of individual mitochondria from CBZ-treated (*red*) or control (*black*) cells, matched for similar fluorescence levels after ionomycin addition (F_iono_; *arrow*) showing lower resting mitochondrial calcium levels after treatment with CBZ. (ii) Normalized mitochondrial calcium levels (F/F_iono_) from cells treated with CBZ (*red*) or vehicle alone (*white*). *P*-values, unpaired Student's *t*-test (*n *≥* *20) (compared to vehicle alone): 9.5 × 10^−15^.

CBZ treatment of macrophages results in (E) a decrease in cellular ATP and consequently (F) increased phosphorylation of AMP kinase (AMPK) and ULK1 (ULK). *P*-values, unpaired Student's *t*-test (*n = *3) (compared to vehicle alone): 0.00003.

Model for mechanism of autophagy induction by CBZ. Inhibition of the Na^+^ channel SCN5A leads to reduced activity of the Na^+^–inositol co-transported SLC5A3 leading to a drop in cellular inositol levels. The subsequent reduction in PIP_2_ (4,5) leads to a fall in basal IP_3_ (1,4,5) and reduced mitochondrial Ca^2+^ levels. The resultant fall in cellular ATP activates AMP kinase and subsequently ULK1 to trigger autophagic disposal of mycobacterial killing. Data information: * *P *< 0.05; ** *P *< 0.005. All experiments were carried out at least in triplicate and on at least three separate occasions.

## Discussion

Through mammalian cell-based screening, we have identified FDA-approved drugs that enhance autophagic killing of mycobacteria and could rapidly be reprofiled for clinical use. Our lead compound, the anticonvulsant carbamazepine, is effective against MDR-TB infection *in vivo* and thus provides first proof-of-principal that pharmacological induction of mTOR-independent autophagy can be used to treat multidrug-resistant mycobacteria. Our finding that carbamazepine enhances autophagy through cellular myo-inositol depletion, while suggesting new mechanisms by which it may act in psychiatric disorders (through altered inositol phosphate signalling (Berridge *et al*, 1989; Harwood, 2005) and/or autophagic regulation of presynaptic neurotransmission (Hernandez *et al*, 2012)), reveals a fundamental role for inositol metabolism in triggering autophagy and has identified a novel, potentially druggable, pathway regulating autophagy induction in macrophages.

Although carbamazepine was capable of enhancing autophagic killing by macrophages infected with all strains of drug-sensitive and drug-resistant mycobacteria tested, we did observe differential, strain-specific effects when treating *M. tuberculosis*-infected mice. Further work will be required to establish whether these treatment failures reflect inadequate dosing of carbamazepine (which is both poorly solubilized and susceptible to variable hepatic metabolism) or true strain-specific differences in susceptibility to CBZ-enhanced or autophagy-dependent clearance *in vivo*. Nevertheless, our work provides firm evidence for the potential of therapeutic autophagy enhancement and a starting point for the development of more effective compounds.

In summary, stimulation of autophagy by carbamazepine avoids the immunosuppressive effects of mTOR inhibition seen with rapamycin, promoting both innate and adaptive immune responses to mycobacterial infection, and represents a novel therapeutic strategy unaffected by bacterial resistance to conventional antibiotics. Our work suggests that while *M. tuberculosis* can successfully overcome physiological processes mediating intracellular killing and survive within macrophages, it remains vulnerable to mTOR-independent autophagic clearance.

## Materials and Methods

### *In vitro* mycobacterial infection assays

#### Luminescent reporter strain infection

A validated luminescent reporter strain of *M. bovis* BCG (BCG-*lux*) encoding the Vibrio *lux AB* gene, generated as previously described (Kampmann *et al*, 2000), was used to infect macrophages. Correlation between colony-forming units and luminescence was confirmed prior to experiments. Primary human macrophages (obtained from consented healthy volunteers under Regional NHS Research Ethics Committee approval), or the mouse macrophage cell line RAW 264.7 (purchased from ATCC and regularly tested for mycoplasma), were inoculated with BCG-*lux* (at MOI of 10:1) for 1 h at 37°C, repeatedly washed and then incubated for 24 h at 37°C in the presence of compounds as indicated. Cells were lysed and luminescence was measured as previously described (Renna *et al*, 2011). Experiments were carried out in sextuplicate. Results are representative of three separate experiments.

#### Screening compounds for autophagic killing activity

Two hundred and fourteen compounds with no direct antibiotic activity, which have been used in human without major toxicity, were screened on multiple occasions for their ability to kill intracellular BCG-lux within macrophages (initially the mouse macrophage cell line RAW 264.7). Hits were then tested both on autophagy-null (ATG12 KD) cells (to demonstrate autophagy-dependent killing) and on primary human macrophages, and for their ability to stimulate mTOR-independent autophagy.

#### Infection with *M. tuberculosis*

Primary human macrophages were generated from peripheral blood obtained from healthy consented subjects (approved by Regional NHS Research Ethics Committee) and were infected with *M. tuberculosis* H37Rv (at MOI of 5:1) for 1 h, repeatedly washed and then incubated for times indicated in the presence of compounds as described. Cells were washed, lysed and plated onto Middlebrook 7H11 agar plates (after serial dilution). Colony-forming units were enumerated after incubation at 37°C. Experiments were carried out in triplicate. Results are representative of three separate experiments.

### Zebrafish experiments

Zebrafish experiments were approved by the Local ethical review panel of the Sheffield University Project applications and amendments committee in accordance with UK legislation and were carried out in accordance with Arrive guidelines. Zebrafish (*Danio rerio*, AB strain), sourced in house, were used at 1–2 days post-fertilization and were housed in Home Office-approved aquaria facilities in the Bateson Centre and the Department of Infection and Immunity, University of Sheffield.

#### Bacterial infection

Wild-type Zebrafish were injected with a red fluorescently tagged *M. marinum* strain M (ATCC #BAA-535), containing the pSMT3-Crimson vector (van der Sar *et al*, 2009). Approximately 125 colony-forming units (CFU) of bacteria were injected into the yolk sac circulation valley at 28 hpf as previously described (Benard *et al*, 2012). Larvae were imaged at 120 hpf on a Zeiss LSM510 NLO upright microscope using a Zeiss Epiplan-Neofluar 2.5× objective (NA0.06). The investigator was not blinded to the treatment condition due to the need to separate the infectious agent from the laboratory environment where the fish were sited. Analysis of total fluorescence throughout the larvae was performed using Volocity® software.

#### *In vivo* autophagy assay

RNA for the fluorescent LC3 construct (pDestCMV:RFP.GFP.LC3) was injected into Zebrafish embryos at the one-cell stage. At 1 dpf, selected embryos were dechorionated manually and incubated for 24 h in vehicle-only control (DMSO 0.01%), rapamycin (1 μM) or carbamazepine (50 μM) in the presence of chloroquine (2.5 μM) to delay degradation of autophagosomes. A section of muscle ventral to the dorsal fin was imaged in approximately 25 *z*-planes (2 μm), and autophagosomes were counted in a single composite image of all z-planes. The investigator was blinded to the treatment condition. A laboratory member not associated with this project used a coding system unknown to the observer. The data are expressed as the mean + SEM. *n* = at least 15, performed as 3 independent experiments. See Supplementary Methods for details.

### Mouse *in vivo* infection model

Specific-pathogen-free female C57BL/6 mice were challenged with multidrug-resistant (MDR) *M. tuberculosis* clinical strains CSU87 (which is resistant to all first line anti-mycobacterial antibiotics) or CSU39 (a less virulent isolate) or a clinical drug-sensitive strain (SA310) using a Glass-Col (Terre Haute, Inc.) aerosol generator calibrated to deliver 50–100 bacteria into the lungs of each mouse as previously described (Ordway *et al*, 2006, 2008). On Day 1 after infection, enumeration of bacteria was performed on two mice. Treatment was started from Day 20 to Day 49 after infection and consisted of the following groups: Control (saline; 0.2 ml per mouse by gavage once daily); RIF/INH (rifampicin 10 mg/kg, isoniazid 25 mg/kg; by gavage once daily); and CBZ (carbamazepine 50 mg/kg; i.p. once daily). On days 20, 35 and 50 following infection, bacterial loads in the lungs and spleen, lung and spleen histology, and flow cytometry were determined in 5 mice from each group. Bacterial counts were determined by plating serial dilutions of homogenates of lungs on nutrient 7H11 agar and counting colony-forming units after incubation at 37°C. Histology and flow cytometry analysis was performed as previously described (Ordway *et al*, 2006, 2008; see Supplementary Methods). All experimental protocols were approved by the Animal Care and Usage Committee of Colorado State University, and experiments were performed in accordance with NIH guidelines. To minimize bias, two groups of independent researchers performed the experiment. One group dosed the animals, whereas the second group determined bacterial burden in the different organs. Lesion scores were determined in a blinded fashion.

### Autophagy analysis in mammalian cell culture

Endogenous LC3-II levels, which directly correlate with autophagosome numbers (Kabeya *et al*, 2000), were detected with anti-LC3 antibody and densitometric analysis, where performed, which is expressed relative to actin. To assess autophagic flux, LC3-II was measured in the presence of 400 nM bafilomycin A1 (treated in the last 4 h), which clamps LC3-II/autophagosome degradation (Klionsky *et al*, 2008). This assay has been established and validated previously with various autophagy modulators (Sarkar *et al*, 2007). Clearance of mutant A53T α-synuclein was performed as previously described (Sarkar *et al*, 2007; see Supplementary Methods).

### Confocal immunofluorescence

Primary human macrophages were grown on glass coverslips, treated as described, rinsed with PBS, fixed with methanol and permeabilized with 0.1% Triton X-100 (Sigma) before incubation with primary and then secondary antibodies as previous described (Floto *et al*, 2005, 2006).

### Myo-inositol effects on autophagosome formation

Raw 264.7 TL macrophages were seeded on glass-bottomed culture dishes at 0.3 × 10^6^ per well (Matek). They were then incubated for 24 h in RPMI 1640, with no myo-inositol, normal media (containing 200 μM of myo-inositol) or excess myo-inositol (5 mM), with or without CBZ (50 μM). They were then imaged under the LSM780, and autophagosome numbers were quantified using Volocity software.

### *Dictyostelium* infection model

Wild-type and mutant *Dictyostelium discoideum* AX2 strains were grown axenically, resuspended in KK2 buffer and incubated with *M. abscessus* (ATCC 1997) expressing *Lux AB* as previously described (Renna *et al*, 2011) at an MOI of 0.01:1 for 2 h. Viable mycobacteria were quantified by measuring luminescence as previously described (Renna *et al*, 2011). Experiments were performed in sextuplicate on at least 3 separate occasions. See Supplementary Methods for details.

### Measurements of myo-inositol uptake, levels of inositol 1,4,5-trisphosphate and phosphatidylinositol bisphosphate

#### Myo-inositol uptake assay

RAW 264.7 cells were treated with carbamazepine (CBZ 200 μM), or vehicle control (DMSO) for 3 ½ h in standard culture media before then being resuspended in flux media (143 mM NaCl, 5 mM KCl, 2 mM CaCl_2_, 1.2 mM MgCl_2_, 10 mM HEPES; pH 7.4) supplemented with 0.02 mM myo-inositol with or without CBZ for 30 min. Cells were then resuspended in fresh flux media or sodium-free flux media (143 mM N-methyl-d-glutamine (NMDG), 5 mM KCl, 2 mM CaCl_2_, 1.2 mM MgCl_2_, 10 mM HEPES; pH 7.4) containing 1 μCi of ^3^H myo-inositol (PerkinElmer) and incubated at 37°C for times indicated before being washed in ice-cold PBS and lysed in 0.25 M NaOH overnight at 4°C. Cell-associated radiation was quantified by scintillation.

#### Quantification of phosphatidylinositol bisphosphate (PIP_2_)

RAW 264.7 cells were treated with carbamazepine (200 μM), or vehicle control (DMSO) for 24 h, the media replaced with ice-cold 1 M HCl. Following chloroform/methanol extraction and phosphate methylation, PIP_2_ species were quantified by HPLC-MS as previously described (Clark *et al*, 2011).

#### Inositol 1,4,5-trisphosphate (IP_3_ 1,4,5) measurements

RAW 264.7 cells were treated with carbamazepine (200 μM), lithium chloride (10 mM) or vehicle control (DMSO) for 48 h, washed with fresh media (and compounds) for 5 min, then scraped, spun and resuspended in ice-cold PBS. Following trichloroacetic acid/1,1,2-trichloro-1,2,2-trifluoroethane/trioctylamine extraction, IP_3_ were measured using the inositol (1,4,5) trisphosphate ^3^H radioreceptor assay kit (PerkinElmer Life Sciences) following manufacturer's instructions.

### Mitochondrial Ca^2+^ measurements

HeLa cells stably expressing mt-cameleon were seeded onto uncoated 35-mm glass-bottomed dishes (MatTek Corporation), grown to 40–70% confluence then treated with 50 μM carbamazepine or vehicle alone for 24 h. Shortly, before imaging, cells were rinsed twice and maintained in calcium containing Hanks' balanced salt solution (HBSS, Gibco) supplemented with 1 mM HEPES (Sigma) and 20% glucose at 37 °C. Fluorescence imaging was achieved as previously described (Tolhurst *et al*, 2011), based on Palmer *et al* (2006). Excitation was achieved at 435 ± 10 nm using a 75-W xenon arc lamp and a monochromator (Cairn Research) controlled by MetaFluor software (Molecular Devices). CFP and YFP emissions were separated using a Cairn Optosplit II Image splitter and acquired with an Orca-ER digital camera (Hamamatsu) at 470 ± 24 and 535 ± 30 nm, respectively. Cells were excited every 5 s, and fluorescence was recorded from individual mitochondria (30–35 in each experiment), background-corrected and expressed as the ratio of CFP over YFP. Once fluorescence signals had stabilized and the temperature equilibrated, cells were imaged at rest for at least 5 min (*R*_basal_) before 10 μM ionomycin/10 mM CaCl_2_ was added to the bath to achieve a reference fluorescence value (*R*_iono_) by which separate experiments could be compared (expressed as *R*_basal_/*R*_iono_). Experiments were carried out on at least three separate occasions and with 3 or more individual wells per condition.

### ATP measurement

Primary human macrophages were cultured in white flat-bottom medium-binding 96-well plates (Greiner Bio; seeding density: 3 × 10^4^/well) and were treated with carbamazepine (50 μM for 24 h, supplemented with fresh media for the last 20 min) or vehicle (DMSO). Cellular ATP concentrations (measured in triplicate independent samples) were assayed using CellTiter Glow (Promega) following the manufacturer's instructions. Experiments were performed on at least three separate occasions.

### Statistics

Sample sizes for *in vitro* experiments were estimated based on previous experience of similar assays and the effect size observed in preliminary experiments. No samples were excluded from analysis. All samples were genetically identical prior to allocation of treatments.

For the assay of autophagy induction in zebrafish, sample size was calculated from experimental data giving a standard deviation of 1,040 autophagosomes and an expected meaningful difference of 1,100 autophagosomes. For 80% confidence, this requires at least 14 per group (*n* = 2 × 7.9 × SD^2^/D^2^). This was performed as three independent experiments with 5 larvae in each group, giving a total of 15 larvae per condition. Animals had to be healthy and express the RNA construct to be included in analysis. These were prespecified. For the assay of *M. marinum* infection burden in zebrafish, sample size was calculated from preliminary data giving a standard deviation of 7.75 arbitrary units and an expected meaningful difference of 10 arbitrary units. For 90% confidence, this requires at least 13 per group (*n* = 2 × 7.9 × SD^2^/D^2^). This was performed as three independent experiments with 5 larvae in each group, giving a total of 15 larvae per condition. No animals were excluded from analysis. Formal randomization was not performed. For the mice experiments, sample size estimates were made based on other similar experiments performed on these (and other) MDR-TB strains and other antimicrobial compounds. No animals were excluded from analysis. For mice experiments, all animals were genetically identical and treatment groups were assigned prior to infection.

Unless where otherwise stated, *P*-values for all assays were determined using two-tailed Student's *t*-test where the data were known or tested (by Sigmaplot statistics package) to be normally distributed. Where appropriate, individual data points were also plotted out to confirm a normal distribution. A *P*-value of 0.05 or less was considered significant. Experiments were performed on at least three separate occasions with at least triplicate samples for each condition and represented as mean ± standard deviation (sd) or standard error (se). All experiments conformed to the principles set out in the WMA Declaration of Helsinki and the Department of Health and Human Services Belmont Report.

## The paper explained

### Problem

The increase in infections with multidrug-resistant (MDR) and extensive drug-resistant (XDR) MTB, requiring longer and more toxic drug regimens which often fail, has created significant barriers to effective treatment of tuberculosis in both well-resourced and resource-poor settings (Nathanson *et al*, 2010; Lienhardt *et al*, 2012) and illustrates the pressing need for new, more effective therapies.

### Results

Here, we show that screening of FDA-approved drugs identified compounds able to stimulate autophagic killing of mycobacteria at therapeutic concentrations. One such compound, carbamazepine, triggers autophagy independently of mTOR (revealing a novel myo-inositol-dependent activation pathway) and effectively targets multidrug-resistant *M. tuberculosis in vivo* stimulating both innate and adaptive immunity. We also demonstrate that *in vivo* autophagic killing of *M. tuberculosis* by carbamazepine is strain-specific, suggesting that autophagy resistance factors may have developed in certain clinical isolates.

### Impact

Pharmacological stimulation of autophagy by carbamazepine avoids the immunosuppressive effects of mTOR inhibition seen with rapamycin, promoting both innate and adaptive immune responses to mycobacterial infection, and represents proof of concept for a novel therapeutic strategy unaffected by bacterial resistance to conventional antibiotics. Our work suggests that while *M. tuberculosis* can successfully overcome physiological processes mediating intracellular killing and survive within macrophages, it remains vulnerable to mTOR-independent autophagic clearance.

## Supporting Information

Supplementary Information

Review Process File
