# Supplementary material for: Functional drug screening reveals anticonvulsants as enhancers of mTOR-independent autophagic killing of Mycobacterium tuberculosis through inositol depletion
Source: EMBO Mol Med. 2014 Dec 22;7(2):127–39. doi: 10.15252/emmm.201404137 (PMC4328644; doi:10.15252/emmm.201404137)
Supplement: Supplementary file 1 [file emmm0007-0127-sd1.pdf]

Functional drug screening reveals anticonvulsants as enhancers of mTOR-independent autophagic killing of *Mycobacterium tuberculosis* through inositol depletion.

Mark Schiebler[1,9], Karen Brown[1,2,9], Krisztina Hegyi[1,9], Sandra M. Newton[3,9], Maurizio Renna[4,9], Lucy Hepburn[1], Catherine Schaffner[1], Sarah Coulter[1], Andres Obregón-Henao[5], Marcela Henao Tamayo[5], Randy Basaraba[5], , Beate Kampmann[3], Katherine M. Henry[6], Joseph Burgon[6], Stephen A. Renshaw[6], Angeleen Fleming[4], Robert R. Kay[7], Karen E. Anderson[8], Phillip T. Hawkins[8], Diane J. Ordway[5], David C. Rubinsztein[4] & R. Andres Floto[1,2].

***Supplementary Material*.**

Included are:

Supplementary Figure (1-9)

Supplementary Table (1)

Supplementary Methods

SUPPLEMENTARY FIGURES

Supplementary Figure 1. Effect of carbamazepine treatment on viability of primary human macrophages. We monitored the effect of 24 h treatment with Carbamazepine (CBZ) on the viability (Annexin V negative, PI negative) of primary human macrophages by flow cytometry (expressed as a percent of values using vehicle alone). Results are representative of 3 independent experiments.

Supplementary Figure 2. Effect of CBZ treatment of mice infected with different clinical isolates of *M. tuberculosis.* Mice were infected with the MDR strain CSU87, or CSU 39, or the drug sensitive strain SA310 and then treated with CBZ (*black*) or vehicle alone (*white*). Colony forming units (CFU) in lung were measured at day 52 post infection. N = 5 per group.

Supplementary Figure 3. Effect of CBZ on lung CD4+ cells

Flow cytometric quantification of $CD4^+$ T cell subsets from lungs of mice infected with the MDR strain CSU 87 as above. Carbamazepine treatment was associated with increases in central memory ($CD4^+$ $CD44^{hi}$ $CD62L^{hi}$) but not effector memory ($CD4^+$ $CD44^{hi}$ $CD62L^{lo}$) T cells, an increase in $IFN\gamma^+$ T cells and a reduction in regulatory ($FoxP3^+$, $IL\text{-}10^+$) T cells. P values in C & D comparing MDR-TB infected, untreated values to MDR-TB infected, CBZ treated values. *N* = 3 mice per group.

Supplementary Figure 4. SCN5A expression in primary human macrophages.

(A) Representative confocal fluorescence images of SCN5A expression in primary human macrophages. **(B)** Images of permeabilised primary human macrophages following siRNA knockdown of SCN5A (siSCN5A; bottom panels) or control siRNA

(siControl; top panels). Cells were stained with SCN5A-specific antibody (SCN5A; *green*) or isotype control antibody (Isotype; *green*) as indicated and the nuclear stain DAPI (*blue*). *N = 3* performed on at least 3 separate occasions.

Supplementary Figure 5. Macrophage viability of inhibitor and CBZ treatment. There is no significant viability effect on infected primary human macrophages, when treated with SCN5A inhibitors or CBZ. *N = 3* performed on at least 3 separate occasions

Supplementary Figure 6. SLC5A3 siRNA knockdown increases autophagosome formation. Knockdown of SLC5A3 in RAW 264.7 macrophages expressing mCherry LC3 induced a significant increase in autophagosome formation. Performed in triplicate ($n \geq$ 30 fields of view analysed per condition) on at least 3 separate occasions. *P* value, unpaired student T test (compared to siControl): 0.000068.

Supplementary Figure 7. LC3 changes in response to myo-inositol depletion. (A) Myo-inositol depletion enhances LC3-II formation, with no difference seen when treating with CBZ (50 μM, 200 μM). LC3-II was however increased when CBZ (50 μM, 200 μM) was added in normal media containing 200 μM of myo-inositol. In excess myo-inositol (5mM) there is no increase in autophagosome formation, regardless of treatment. **(B)** Clearance of the known autophagy substrate (A53T) α-synuclein in stable inducible PC12 cells was assessed by western blot. As expected, the autophagy enhancer rapamycin (Rap) enhanced clearance, while the known autophagy inhibitor forskolin (FSK) blocked clearance of A53T α- synuclein. Incubation of cells in myoinositol-free media enhanced clearance of A53T α- synuclein, which was not further increased by treatment with carbamazepine (CBZ, 50 μM). *N* = 3.

Supplementary Figure 8. Effect of autophagy inhibitors on killing of *M. abscessus* by *Dictyostelium.* Wild type *Dictyostelium* AX2 was grown axenically and incubated with *M. abscessus-lux* (Renna *et al*., 2011) at an MOI of 0.01:1. *M. abscessus-lux* with no *Dictyostelium* (*white*) were grown under identical conditions as a control. Viable mycobacteria were quantified by measuring luminescence at 2 hours post infection in the presence of inhibitors of autophagy: 3MA (5 mM), wortmannin (20 nM), chloroquine (10 μM), or NH_4Cl (1 mM). *N = 6* performed on at least 3 separate occasions. *P* values, unpaired student T test (compared to *Dictyostelium* alone): 3MA 0.014; Wortmannin 0.000065; Chloroquine 0.0000463; NH_4Cl 0.00012.

Supplementary Figure 9. CBZ treatment increases P-AMPK in infected macrophages. CBZ and VPA treatment both increase the ratio of P-AMPK to AMPK in infected primary human macrophages. *N* = 3.

Supplementary Table 1. MIC of conventional and novel anti-tuberculosis

Neither CBZ nor VPA have a direct inhibitory growth effect at any of the concentrations measured on any mycobacterial strain tested. Conventional anti-TB drugs Moxifloxacin and Rifampicin exhibit an MIC on both M. bovis BCG and drug susceptible M.TB (H37RV) within the range treated. As expected Rifampicin has no inhibitory effect on the multi-drug resistant strain (CSU-87).

SUPPLEMENTARY METHODS

Constructs, antibodies and chemicals.

Constructs and siRNA: The mitochondrial Ca^{2+}-sensor mt-cameleon (2mtD2cpv) was a kind gift of Roger Tsien (*1*). SiRNA targeting Atg12, SLC5A3, SCN5A and non-targeting control (siGENOME SMARTpool; Dharmacon) were transfected into primary human macrophages using HiPerfect (Qiagen) following manufacturer's instructions. The final siRNA concentration was 100 nM. Cells were used for experiments after 3 days. The knockdown efficiency was assessed by Western blot and RT-PCR. The level of ATG12 protein knockdown achieved was at least 95%. The level of SLC5A3 achieved (by RT-PCR) was between 80 and 83%.

Antibodies: The following antibodies were used: anti-actin polyclonal (Sigma; A2066); anti-LC3 (Novus Biological or Nano-Tools NB100-2220); monoclonal anti-HA (Covance; Clone 16B12); polyclonal anti-phospho and total p70S6kinase and anti-phospho and total S6 ribosomal protein antibodies (Cell Signaling; 9205, 9202, 2215, 2217); Rab7-specific antibody (Cell Signaling; 9367); ATG12-specific antibody (Cell Signaling; 2011); SCN5A-specific antibody (Alomone labs; ASC-005); phospho-specific and total AMPKinase (Cell Signaling; 2535, 2532); phospho-specific and total ULK1 (Cell Signaling; 6888, 4773); anti-mouse/rabbit HRP-conjugated secondary antibodies (GE Healthcare; 25005174, 25005179).

Chemicals: The following chemicals were used (solvents and providers in brackets): carbamazepine (DMSO, Sigma), valproic acid (DMSO, Sigma), bafilomycin A1 (DMSO, Millipore), rapamycin (DMSO, Sigma), 4',6-diamidino-2-phenylindole (Sigma), doxycycline (Sigma), Interferon-γ (Peprotech). Unless otherwise stated, other laboratory chemicals were purchased from Sigma.

Culture of cell lines and isolation of human macrophages

Cell lines: HeLa and COS-7 were grown in DMEM supplemented with 10 % FBS, 100 U/ml penicillin/streptomycin, 2 mM L-glutamine at 37 °C with the addition of G418 (600 μg/ml) for HeLa cells stably expressing the mitochondrial Ca^{2+}-sensor mt-cameleon. The PC12 A53T α-syn Tet-on inducible cell line (*2*) was maintained in DMEM, 10 % horse serum, 5 % FBS, 100 U/ml penicillin/ streptomycin, 2 mM L-glutamine, 75 μg/ml hygromycin B and 100 μg/ml G418.

Primary human cells: Human monocyte-derived macrophages were generated as previously described (*3*). Briefly Monocytes were extracted by $CD14^{+}$ positive selection from PBMC using magnetic beads (Miltenyi) and differentiated into macrophages by 5-10 day culture in RPMI 1640, 10 % FCS, 100 U/ml penicillin/streptomycin, 2 mM L-glutamine supplemented with 400 ng/ml M-CSF (Peprotech). Non-adherent cells were discarded prior to experiments. Human alveolar macrophages were isolated by adherence from bronchoalveolar lavage fluid obtained from consented individuals undergoing diagnostic bronchoscopy (approved by Regional NHS Research Ethics Committee) and cultured as above.

Macrophage viability

Primary human macrophages were seeded at 1 x10^6 per well in a 6 well plate. Once differentiated they were infected at an MOI of 10:1, as previously described. After infection they were treated with CBZ, Opripramol, lofepramine, trimipramine, mianserin, flecainide at cocentrations previously indicated (**Fig 4B**). 1 μL/ml of SYTOX AADvanced dead cell stain solution in DMSO was added to each flow cytometer sample. The samples were then protected from light and incubated for 5 minutes at room temperature before being analysed using the 488 nM excitation and emission collected at 695/40 nM.

Effects of myo-inositol concentration on LC3.

Raw 264.7 TL macrophages were seeded on glass bottomed culture dishes at 0.3 x10^6 per well (Matek). They were then imaged under the LSM780 and autophagosome numbers were quantified using velocity. For western blotting RAW 264.7 macrophages were seeded on 6 well plates. They were then incubated for 24 h in RPMI 1640, with no myo-inositol, normal media (containing 200μM of myo-inositol) or excess myo-inositol (5mM), with or without CBZ (50 μM,200 μM), after which lysates were taken.

P-AMPK in infected macrophages treated with CBZ.

Primary human macrophages infected with *Mycobacterium bovis* were treated with Carbamazepine (50 μM), and then after 24 h the lysates were taken and AMPK, P-AMPK levels were measured.

SLC5A siRNA knockdown effects on autophagosome formation

Raw 264.7 TL macrophages seeded on glass bottomed culture dishes at 0.3 x10^6 per well (Matek). They were then knocked down with either SI control, or SLC5A siRNA for 72 h, where they were imaged under the LSM780 and autophagosome numbers were quantified using velocity.

Autophagy analysis in mammalian cell culture

Endogenous LC3-II levels, which directly correlate with autophagosome numbers (*4*), were detected with anti-LC3 antibody and densitometric analysis, where performed, is expressed relative to actin. To assess autophagic flux, LC3-II was measured in the presence of 400 nM bafilomycin A1 (treated in the last 4 h), which clamps LC3-II/autophagosome degradation (*5*). This assay has been established and validated previously with various autophagy modulators (*2*).

Clearance of mutant A53T α-synuclein

As previously described (*2*), stable inducible PC12 cell lines expressing A53T α-synuclein were induced with 1 μg/ml doxycycline for 48 hours. Transgene expression was switched off by removing the antibiotic from medium and then the cells were treated for 24 hours with compounds or vehicle alone as indicated. For experiments involving myo-inositol removal, cells were resuspended in myo-inositol-free DMEM (custom produced by Millipore) containing dialysed 5 % FBS and 10 % horse serum. The levels of A53T transgene were then assessed by western blot analysis. Experiments were performed in triplicate and on at least three different occasions and quantified by densitometry normalized to actin.

Confocal immunofluorescence

Primary human macrophages were grown on glass coverslips, treated as described, rinsed with PBS, fixed with methanol and permeabilized with 0.1% Triton-X100 (Sigma) before incubation with primary and then secondary antibodies as previous described (*6*). Cells were washed between antibody incubations and rinsed with water before mounting with ProLong Gold antifade containing DAPI (Invitrogen). Images were

acquired on a Zeiss LSM710 confocal microscope (Plan-Apochromat 63x/1.40 Oil immersion lens) and analysed with Zen 2010 software and Zeiss LSM Image Browser (Carl Zeiss).

ATP measurement

Primary human macrophages were cultured in white flat bottom medium-binding 96-well plates (Greiner Bio; seeding density: 3 x 10^4/well) and were treated with carbamazepine (50 μM for 24h, supplemented with fresh media for the last 20 min) or vehicle (DMSO). Cellular ATP concentrations (measured in triplicate independent samples) were assayed using CellTiter Glow (Promega) following the manufacturer's instructions. Experiments were performed on at least 3 separate occasions.

Dictyostelium infection model

Wild type and mutant *Dictyostelium discoidium* AX2 strains were grown axenically (*11*) using HL5 plus glucose (Formedia) in 10 cm plastic Petri dishes or in shaking suspension at 22°C. Cells were also maintained on bacterial *Klebsiella aerogenes* lawns on SM agar plates (*12*). The following mutants (obtained from DictyBase or from the Kay laboratory) were used: *mipp1*- (multiple inositol polyphosphate phosphatase null; *Ref. 13*); I6KA- (inositol hexakisphophate phosphate kinase null; *Ref. 14*); plc- (phospholipase C null; *Ref. 15*); iplA- (inositol (1,4,5) trisphosphate receptor null; *Ref. 16*).

For mycobacterial infection assays, cells were resuspendend in KK2 buffer (16.5 mM KH_2PO_4, 3.9 mM K_2HPO_4, and 2 mM $MgSO_4$) with 0.1 mM $CaCl_2$ and plated onto 1.8% (wt/vol) non-nutrient agar plates at 22°C and incubated with *M. abscessus* (ATCC 19977) expressing *Lux AB* as previously described (*10*) at an MOI of 0.01:1 for 2 hours. Viable mycobacteria were quantified by measuring luminescence as previously described (*10*).

Mouse in vivo infection model

Infection and treatment of mice: Mice were challenged with multi-drug resistant (MDR) *M. tuberculosis* strains CSU87 (which is resistant to all first line anti-mycobacterial antibiotics;) using a Glas-Col (Terre Haute, Inc.) aerosol generator calibrated to deliver 50-100 bacteria into the lungs of each mouse as previously described (Ordway et al., 2006; Ordway et al., 2008). The bacterial strains used have been characterized in previous publications: CSU 87 (Parks *et al.,* 2006); CSU39 (Rhodes & Orme 1997); CSUSA310 (Palanisamy *et al.,* 2009).

On Day 1 after infection enumeration of bacteria was performed on two mice. Treatment was started from Day 20 to Day 49 after infection and consisted of the following groups: Control (saline; 0.2 ml per mouse by gavage once daily); RIF/INH (rifampicin 10 mg/kg, isoniazid 25 mg/kg; by gavage once daily); CBZ (carbamazepine 50 mg/kg; i.p once daily). On Day 20, 35, and 50 following infection, bacterial loads in the lungs and spleen, lung and spleen histology, and flow cytometry were determined in 5 mice from each group. Bacterial counts were determined by plating serial dilutions of homogenates of lungs on nutrient 7H11 agar and counting colony-forming units after incubation at 37^oC.

Histological analysis: Lung tissues were collected for histopathological analysis on days 20, 35, and 50 following of the infection. The accessory lobe of the lung was placed in 4% paraformaldehyde in phosphate buffered saline (PBS) for fixation. Paraffin blocks were then prepared and sections were cut and stained with hematoxylin and eosin (H&E) as previously described (*20, 21*).

Flow Cytometry for surface markers and intracellular cytokines: Mice were euthanized by CO_2 asphyxiation, and the thoracic cavity was opened. Single cell suspension were

prepared as previously described (*20, 21*). Briefly, the lungs were perfused with PBS containing heparin (50 U/ml; Sigma-Aldrich, St. Louis, MO) through the pulmonary artery and aseptically removed from the pulmonary cavity, and dissected in media. Spleens and pulmonary lymph nodes (LN) were also harvested. The dissected tissue was further incubated with DMEM media containing collagenase XI (0.7 mg/ml; Sigma-Aldrich) and type IV bovine pancreatic DNase (30 μg/ml; Sigma-Aldrich) for 30 minutes at 37°C. The digested lungs were then disrupted by gently pushing the tissue through a cell strainer (BD Biosciences, Lincoln Park, NJ). Red blood cells were lysed with ACK buffer, washed and resuspended in complete DMEM containing 10% fetal bovine serum (ATLAS biologicals, CO). Total cell numbers were determined by flow cytometry using BD™ Liquid Counting Beads, as described by the manufacturer (BD PharMingen, San Jose, CA). Cell suspensions from each individual mouse were incubated with monoclonal antibodies labeled with different fluorochromes at 4°C for 30 minutes in the dark. mAbs were used against the surface Marker CD4 (clone Gk1.5,rat IgG2b,k), CD8 (clone 53-6.7, rat IgG2a,k), CD25 (PC61.5, Rat IgG1, λ) CD44 (IM7, Rat IgG2b, K), CD62L (MEL-14, Rat IgG2a,K), CCR7 (4B12, Rat IgG2a,k) , CD45RB (16A, rat IgG2a), CD11c (clone HL3, hamster IgG1), CD11b (Mac-1, clone M1/70, rat IgG2a), I-A/I-E MHC class II (clone 2G9, rat IgG2a) and isotype control rat IgG2a, rat IgG2b, rat IgG1, mouse IgG1, and hamster IgG were used in this study. These mAbs were purchased from BD Pharmingen or eBioscience (San Diego, CA) as direct conjugates to FITC, PE, PerCP, APC, eFluor450, Alexa Fluor700 or Qdot800. All the samples were analyzed on a Becton Dickinson LSR-II instrument, and data were analyzed using FACSDiva v5.0.1 software. Cells were gated on lymphocytes based on characteristic forward and side scatter profiles. Individual cell populations were identified according to the presence of specific fluorescence-labeled antibodies. For intracellular cytokine staining lung, lymph node, or spleen cells were stimulated for 4 hours with anti-CD3 and anti-CD28 in the presence of Golgi Stop (Becton Dickinson). After incubation, cells were harvested and first stained for cell surface markers as indicated above, and thereafter the same cell pellets were resuspended in permeabilization buffer using a commercial kit (Foxp3 Staining Buffer Set; eBiosciences) and incubated for 30 min at room temperature. Cells were washed again and resuspended in Perm/Wash buffer containing labelled mAbs against, Foxp3 (FJK-16s, Rat IgG2a), IFN-γ (XMG1.2, rat IgG1), IL-27 (eBio17B3, Rat IgG2a, κ) and IL-12 (eBio22F10, Rat IgG2a), TNF-α (XMG1.1, rat IgG1), and incubated for 30 minutes at 4ºC. The cells were then washed twice and resuspended in PBS containing 0.05% sodium azide prior to analysis. All the analyses were performed with acquisition of a minimum of 200,000 events.

Statistical analysis in mice: Data are presented using the mean values from 4-5 mice per group and from values from replicate samples. The Student t-test test was used to assess statistical significance between groups of data.

SUPPLEMENTARY REFERENCES

1. Palmer AE et al. Ca2+ indicators based on computationally redesigned calmodulin-peptide pairs. *Chem Biol.* 2006; 13: 521-530.

2. Sarkar S, Ravikumar B, Rubinsztein DC. Autophagic clearance of aggregate-prone proteins associated with neurodegeneration. *Methods Enzymol.* 2007; *453*: 83-110.

3. Floto RA, et al. Loss of function of a lupus-associated FcgammaRIIb polymorphism through exclusion from lipid rafts. *Nat Med.* 2005; 11: 1056-1058.

4. Kabeya Y et al. LC3, a mammalian homologue of yeast Apg8p, is localized in autophagosome membranes after processing. *EMBO J.* 2000: 19, 5720-5728.

5. Klionsky DJ, Elazar Z, Seglen PO, Rubinsztein DC .Does bafilomycin A1 block the fusion of autophagosomes with lysosomes? *Autophagy* 2008; 4: 849-950.

6. Floto R.A, et al. Dendritic cell stimulation by mycobacterial Hsp70 is mediated through CCR5. *Science.* 2006; 314: 454-458.

7. Tolhurst G. et al Glutamine triggers and potentiates glucagon-like peptide-1 secretion by raising cytosolic Ca2+ and cAMP. *Endocrinology.* 2011: 152, 405-413.

8. Clark J et al. Quantification of PtdInsP3 molecular species in cells and tissues by mass spectrometry. *Nat Methods* 2011; 8: 267-272.

9. Kampmann B. et al. Evaluation of human antimycobacterial immunity using recombinant reporter mycobacteria. *J Infect Dis.* 2000: **182**; 895-901.

10. Renna M. et al. Azithromycin blocks autophagy and may predispose cystic fibrosis patients to mycobacterial infection. *J Clin Invest.* 2011; 121: 3554-3563.

11. Watts DJ & Ashworth JM. Growth of myxameobae of the cellular slime mould Dictyostelium discoideum in axenic culture. *Biochem J.* 1970; 119: 171-174.

12. Kay RR. Cell differentiation in monolayers and the investigation of slime mold morphogens. *Methods Cell Biol.* 1987; 28: 433-448.

13. Cho J et al. Dephosphoylation of 2,3-bisphophoglycerate by MIPP expands the regulatory capacity of the Rapoport-Luebering glycolytic shunt. *PNAS*. 2008; 105: 5998-6003.

14. Luo HR et al. Inositol pyrophosphates mediate chemotaxis in Dictyostelium via pleckstrin homology domain-PtdIns(3,4,5)P3 interactions. Cell 2003;114: 559-72.

15. Kortholt A et al. Phospholipase C regulation of phosphatidylinositol 3,4,5-trisphosphate-mediated chomotaxis. MBC 2007; 18: 4772-4779.

16. Traynor D, Milne JL, Insall RH & Kay RR. Ca2+ signaling is not required for chemotaxis in Dictyostelium *EMBO J.* 2000; 19: 4846-4854.

17. Nusslein-Volhard C; Dahm, R. Zebrafish. *A Practical Approach*. 2002

18. van der Sar AM et al. Specificity of the zebrafish host transcriptome response to acute and chronic mycobacterial infection and the role of innate and adaptive immune components. *Mol Immunol.* 2009; 46: 2317–2332.

19. Benard EL et al. Infection of zebrafish embryos with intracellular bacterial pathogens. *J Vis Exp.* 2012; 61: 3781.

20. Ordway D et al. Enhanced macrophage activity in granulomatous lesions of immune mice challenged with Mycobacterium tuberculosis. *J Immunol.* 2006; 176: 4931-4939.

21. Ordway D et al. Animal model of Mycobacterium abscessus lung infection. *J Leukoc Biol.* 2008; 83: 1502-1511.

Supplementary Figure 1

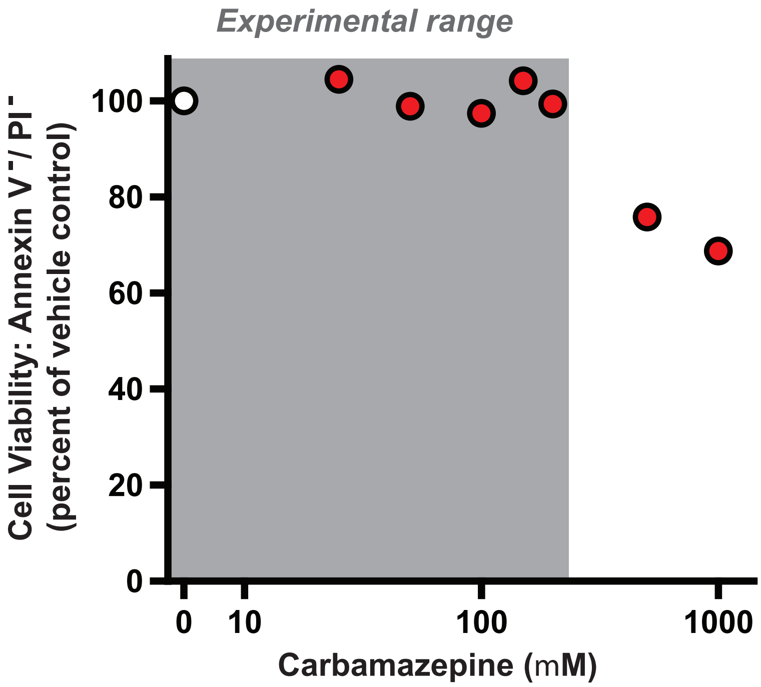

Supplementary Figure 2

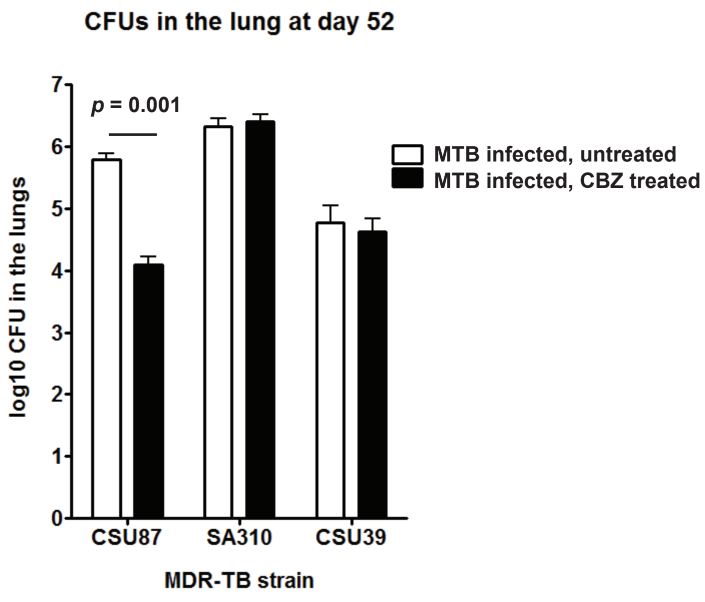

Supplementary Figure 3

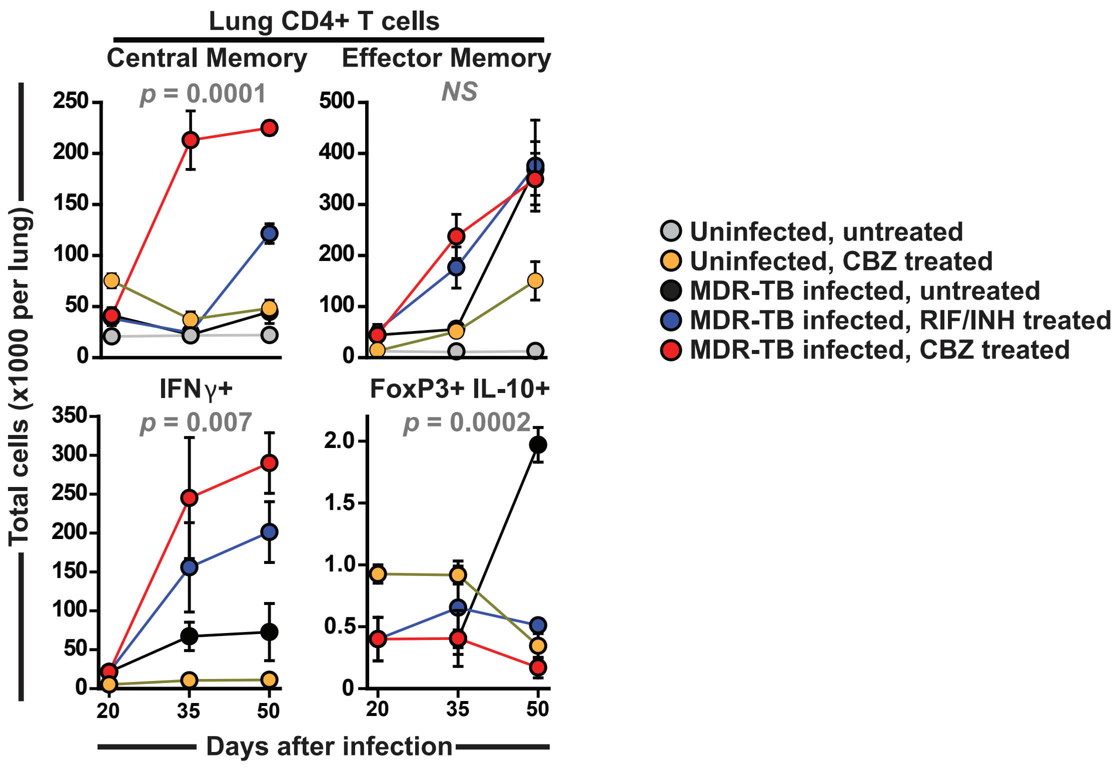

Supplementary Figure 4

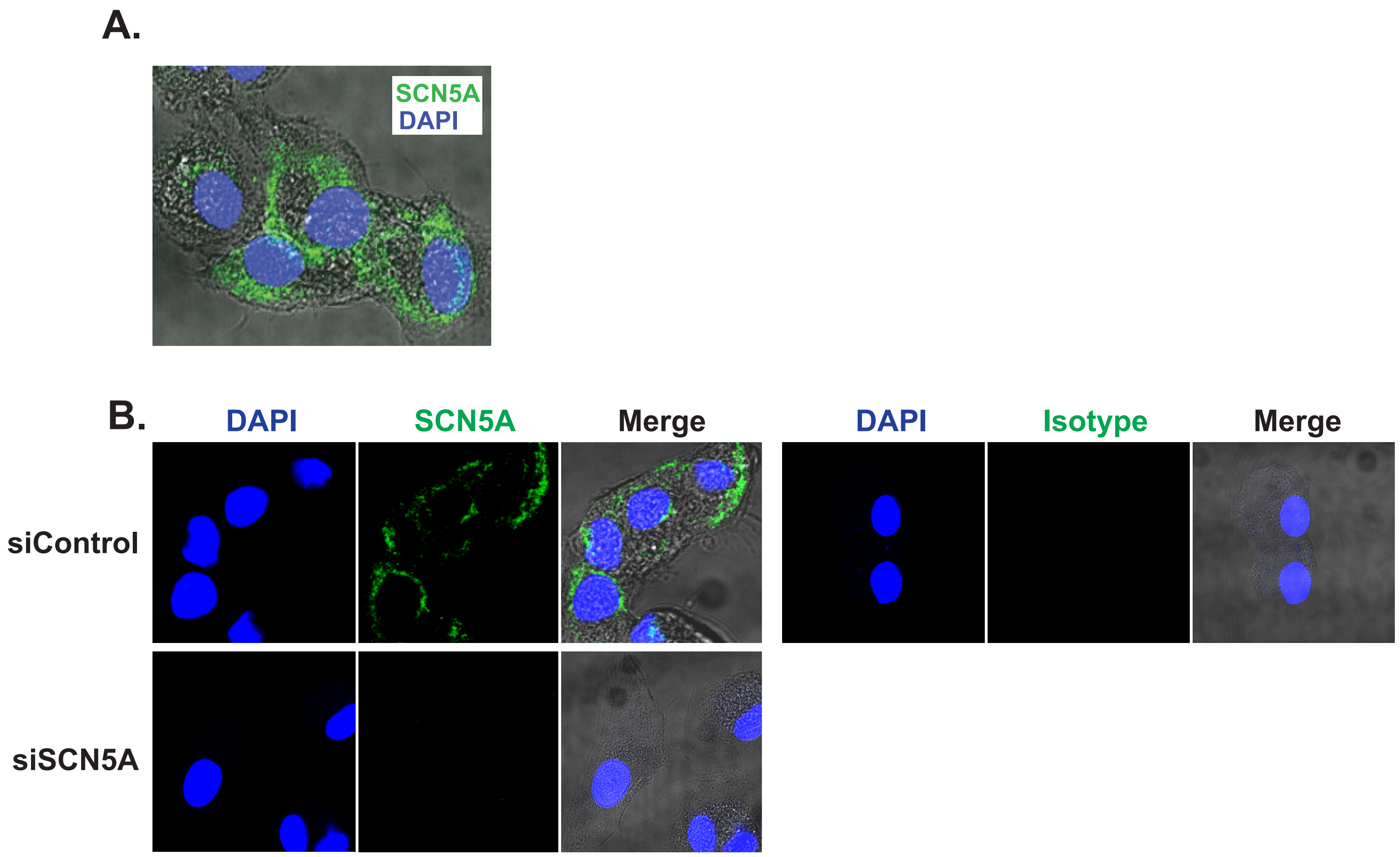

Supplementary Figure 5

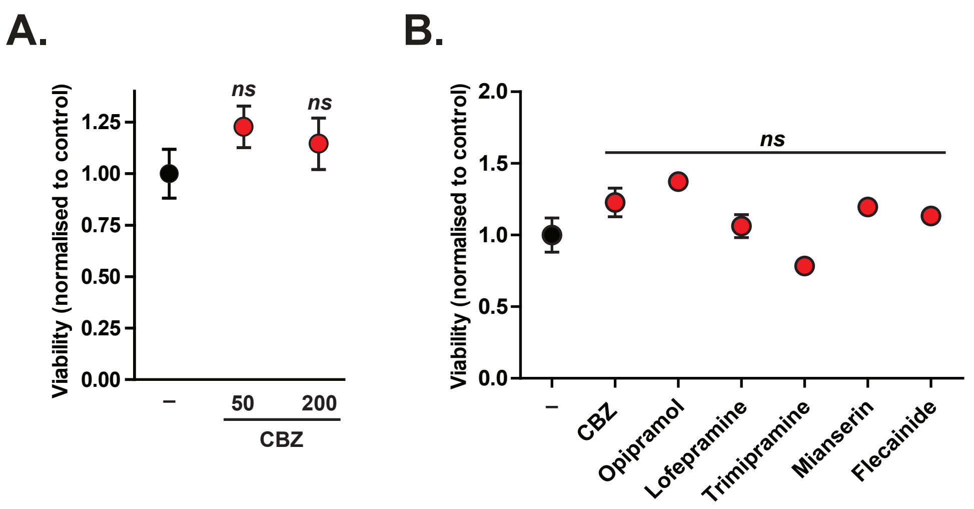

Supplementary Figure 6

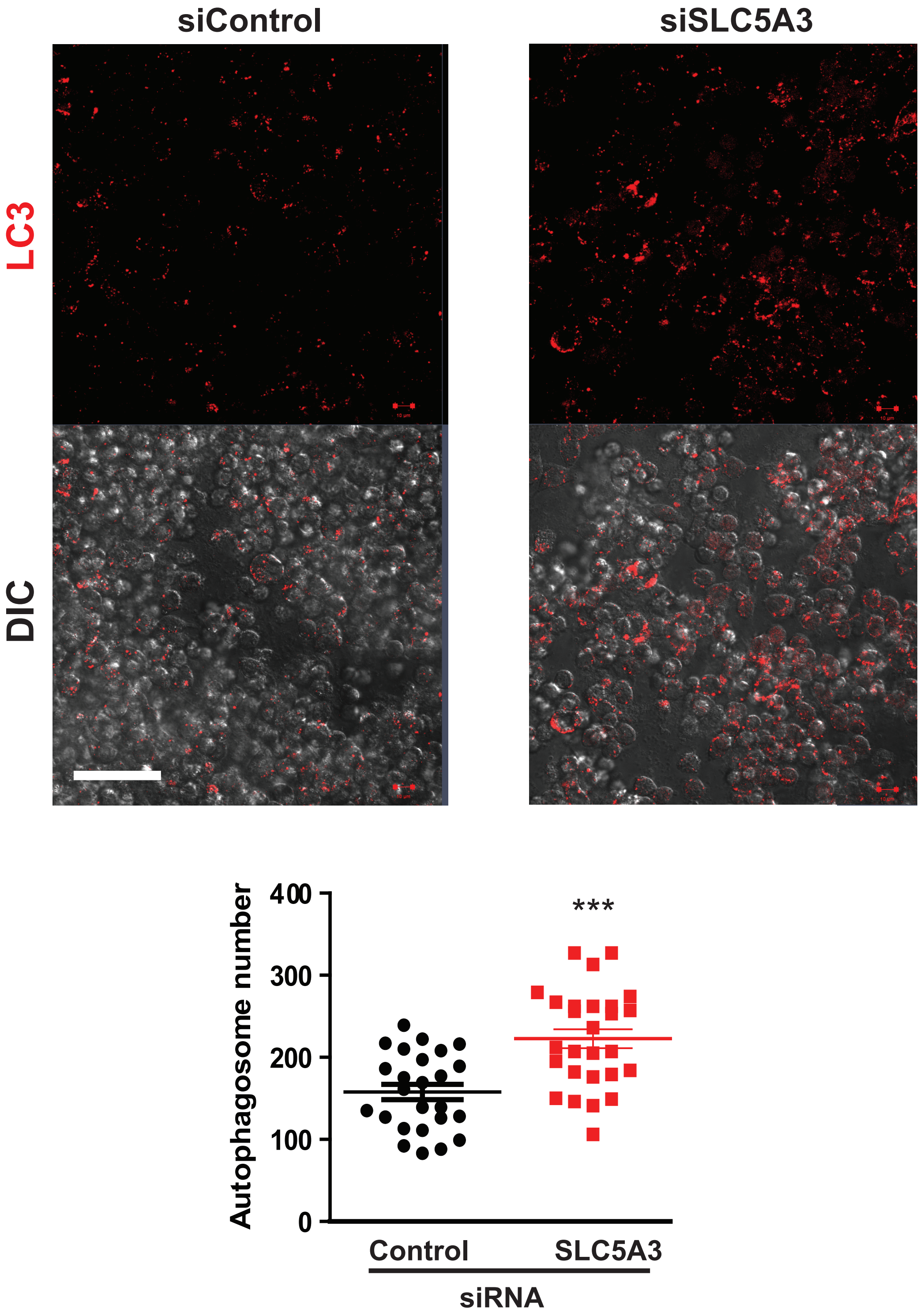

Supplementary Figure 7

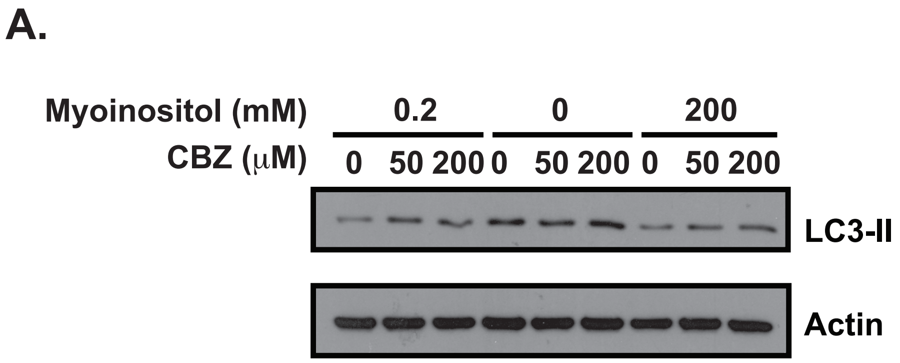

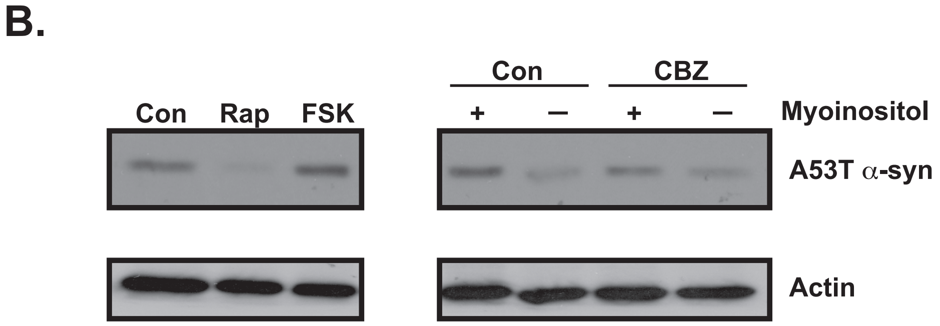

Supplementary Figure 8

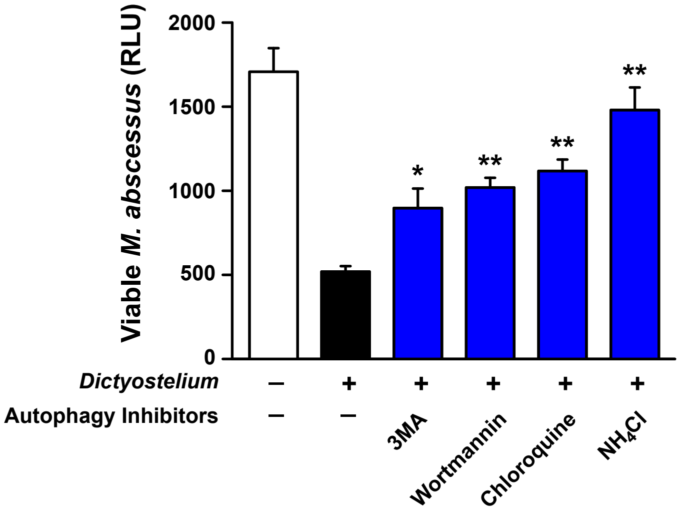

Supplementary Figure 9

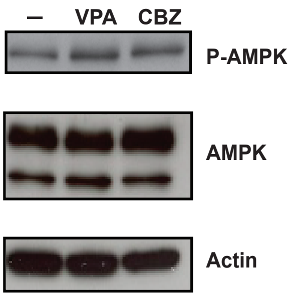

Supplementary Table 1

Antibiotics	Concentration range tested	*M. bovis* BCG	MTB (H37Rv)	MDR-TB (CSU87)
RIF	0 - 16 µg / ml	0.5 µg / ml	0.5 µg / ml	*R*
MOX	0 - 8 µg / ml	0.06 µg / ml	0.06 µg / ml	0.12 µg / ml
CBZ	0 - 400 µM	*R*	*R*	*R*
VPA	0 - 100 µM	*R*	*R*	*R*
