# Supplementary material for: Functional drug screening reveals anticonvulsants as enhancers of mTOR-independent autophagic killing of Mycobacterium tuberculosis through inositol depletion
Source: EMBO Mol Med. 2014 Dec 22;7(2):127–39. doi: 10.15252/emmm.201404137 (PMC4328644; doi:10.15252/emmm.201404137)
Supplement: Supplementary file 2 [file emmm0007-0127-sd2.pdf]

EMBO Molecular Medicine

Functional drug screening reveals anticonvulsants as enhancers of mTOR-independent autophagic killing of Mycobacterium tuberculosis through inositol depletion.

Mark Schiebler, Karen Brown, Krisztina Hegyi, Sandra M. Newton, Maurizio Renna, Lucy Hepburn, Catherine Klapholz, Sarah Coulter, Andres Obregón -Henao, Marcela Henao Tamayo, Randall Basaraba, Beate Kampmann, Katherine M. Henry, Joseph Burgon, Stephen A. Renshaw, Angeleen Fleming, Robert R. Kay, Karen E. Anderson, Phillip T. Hawkins, Diane J. Ordway, David C. Rubinsztein and R. Andres Floto

Corresponding author: Rodrigo Floto, University of Cambridge

Review timeline:

Submission date:	02 April 2014
Editorial Decision:	06 May 2014
Revision received:	11 October 2014
Editorial Decision:	03 November 2014
Revision received:	01 December 2014
Accepted:	02 December 2014

Transaction Report:

(Note: With the exception of the correction of typographical or spelling errors that could be a source of ambiguity, letters and reports are not edited. The original formatting of letters and referee reports may not be reflected in this compilation.)

Editor: Céline Carret

1st Editorial Decision 06 May 2014

Thank you for the submission of your manuscript to EMBO Molecular Medicine. We have now heard back from the three referees whom we asked to evaluate your manuscript. Although the referees find the study to be solid and interesting, they also suggest a number of improvements to make the paper even more compelling and readable.

As you will see from the reports below, all three referees are positive about the study. Nevertheless, all three reviewers find the paper missing in clarity and discussions here and there. Referee 1 also suggests adding some controls and importantly better explaining the effect of CBZ on autophagy. Referee 1 also suggests removing the part where CBZ is tested against different Mtb clinical strains as no mechanism is shown. However, we would like to argue against this suggestion as in our view, this point increases the clinical relevance of the findings; we would not require additional mechanism for this part. Finally, referees 2 and 3 suggest to reorganise the figures in terms of number and panels and referee 3 makes clear suggestions for that.

We would welcome the submission of a revised version for further consideration and depending on the nature of the revisions, this may be sent back to the referees for another round of review.

Please note that it is EMBO Molecular Medicine policy to allow a single round of revision in order to avoid the delayed publication of research findings. Consequently, acceptance or rejection of the manuscript will depend on the completeness of your responses included in the next version of the manuscript.

EMBO Molecular Medicine has a "scooping protection" policy, whereby similar findings that are published by others during review or revision are not a criterion for rejection. Should you decide to submit a revised version, I do ask that you get in touch after three months if you have not completed it, to update us on the status.

Please also contact us as soon as possible if similar work is published elsewhere. If other work is published we may not be able to extend the revision period beyond three months.

I look forward to seeing a revised form of your manuscript as soon as possible.

***** Reviewer's comments *****

Referee #1 (Comments on Novelty/Model System):

The mouse model of Mtb is a well-validated preclinical model.

Referee #1 (Remarks):

The manuscript from Brown et al. describes a novel and compelling new approach to host-directed TB therapy. The major conclusions of the work are that FDA-approved anti-convulsants (particularly carbamazepine, CBZ) can restrict mycobacterial growth through the induction of autophagy. As this process is mTOR independent, it might be a more specific therapy than immunosuppressants such as rapamycin. The potential of this approach is shown in a variety of infection models, most convincingly in the mouse model.

In general, this is a compelling study and represents a huge amount of work. In fact, it would be easy to imagine that this amount of work would be best presented in multiple manuscripts in which each conclusion were more thoroughly supported. Because so much ground is covered, we are compelled to comment on each conclusion and the experiments required to make each scientifically sound. The major conclusions of the manuscript are:

1. CBZ and VPA induce autophagy in mammalian cells to restrict Mtb. This conclusion is generally well supported. The representation/discussion of these experiments could be improved by:
-Explaining the initial drug screen that led to the identification of CBZ and VPA. No information is provided.
-it is unclear how intracellular and extracellular bacteria were differentiated
-In figure 1C, the "growth/nogrowth" metric is unsatisfying. Actual data would be more customary.
-scale bars should be added to the images
-In figure 1I.ii., the ATG12si appears to reduce bacterial numbers to the same degree as CBZ treatment. This suggests that the proper interpretation of the experiment would be that CBZ acts by the inhibition of autophagy, not its activation. This should be addressed or explained.
-Figure 1j should be presented on a log scale, since the bacteria appear to be growing exponentially.
-macrophage cell death in response to compound +/- infection should be assessed.
-Autophagy activation during Mtb infection is not demonstrated. This is important, as the pathways

leading to autophagy have been shown to depend on the ESX secretion system that is deleted in BCG. An LC3 I vs II blot would likely suffice.

2. CBZ activation of autophagy is mTOR independent and targets SCN5A
-Throughout figure 2, western blots for SCN5A expression and knockdown would be more convincing and quantitative than immunofluorescence.
-The authors state that, "We first looked for the presence of known molecular targets of CBZ in primary human macrophages and identified the voltage- and stretch- activated sodium channel SCN5A". It would be useful to know what other targets they looked for. Was SCN5A the only one present?
-as above, controls for macrophage cell viability during inhibitor treatment would be useful.
-while SLC5A knockdown reduces BCG growth, it is not clear that this knockdown also inhibits autophagy. This should be shown in the context of BCG infection.

3. CBZ induces autophagy by reducing IP3 levels and activating AMPK and ULK
-While the biochemical readouts presented in figure 3H-L are consistent with this model, it seems that these experiments were done in uninfected macrophages. If so, infection could change these results. The model would be strengthened if a subset of these measurements were made in infected cells.
-In Figure 2 Fii, the effect of myoinositol on BCG growth is confirmed. A simultaneous demonstration that autophagy is altered would strengthen the model.
-The rationale for using the dictystelium/M. abscessus model is unclear. It is difficult to believe that this is similar enough to the Mtb/macrophage system for the details of the mechanism to be completely complementary. For these data to be meaningful, the authors would have to show that the ultimate mechanism of bacterial control is the same (i.e. show autophagy is required).

4. CBZ treatment activates autophagy in zebrafish and controls M. marinum growth.
-These data are a nice complement to the mouse studies. However, more explanation would help. For example, the LC3 images do not appear representative, as the bar graphs show a much smaller increase in autophagosome number than would be predicted by the images presented.
-do zebrafish have an ortholog for SCN5A?
-The M. marinum growth during rapamycin treatment is not shown. Why? This would be a great comparison with CBZ.

5. CBZ activates autophagy in vivo in mice. This restricts MDR Mtb growth and enhances immunity.
-These data are generally robust and compelling. However, the presentation of the FACS data could be improved in the following ways:
a. CD11b/CD11a markers are insufficient to differentiate macrophages from DC's. These populations should be referred to by their staining characteristics only.
b. Gating strategies should be defined.
c. Total cell numbers might be more informative than % positive cells. If the authors choose not to present the data this way, the total number of leukocytes (aka. The denominator) should also be provided.
d. a brief discussion of the relevance of CM vs EM populations would be helpful.
e. were the lungs profused? This is an important detail to be added to the methods.

5. Different Mtb clinical strains are differentially susceptible to CBZ treatment.
-The LC3 flow cytometry data experiment is interesting, but representative scatter plot should be provided. Also the total cell number (in addition to %) would be useful.
-In panel E the total cfu numbers instead of % reduction should be shown. This is necessary for interpreting panels F and G.
-This is a very interesting observation. The authors rightly state that the mechanism underlying the differential effect of CBZ is unclear. To understand this effect properly, the effect of autophagy would need to be assessed for each strain in cultured macrophages, and additional in vivo work may be required. As a result, the authors should consider removing these data and presenting them in a

separate manuscript once the effect is understood.

Referee #2 (Comments on Novelty/Model System):

Each of the experimental models applied are well established in the microbial pathogenesis field. The next stage of testing would be non-human primates. However, before extending to that model, I would recommend derivatives of the lead compound be tested to optimize its activity.

Referee #2 (Remarks):

In an ambitious study, Brown et al characterize in detail the cellular pathways by which the drug carbamazepine (CPZ) induces autophagy and increases clearance of virulent Mycobacteria from cultured human macrophages, Dictyostelium amoebae, zebrafish, and mice. The authors identify components of the CPZ signaling pathway by applying classical and molecular genetics as well as a logical panel of pharmacological inhibitors in these experimental model systems.

In general, the study is rigorous, quantitative, and thorough. The value of the manuscript is to characterize 1) a non-mTOR-dependent pathway that induces autophagy and 2) a lead compound that modestly reduces the in vivo burden of some but not all virulent strains of Mycobacterium tuberculosis and that is approved for use in humans as a anticonvulsant. The pressing need for clinical treatments to control MDR and XDR M. tuberculosis raises the value of this cell biological study.

To increase the clarity of the manuscript for a broad readership, several recommendations are provided below.

1. The language of the Abstract and Results should more accurately reflect that the impact of CBZ on pathogen clearance is modest (ie ~ 30% reduction in bacterial burden). For example, line 6 of the Abstract implies a sterilizing clearance of infection by CBZ treatment.

2. CBZ treatment itself reports to be pro-inflammatory after long treatment periods (yellow lines in Fig 4C). Please discuss.

3. Multiple cell types are used as hosts, making it challenging for the reader to interpret panel-to-panel differences (eg Fig 1 A vs Fig 1 Iii vs Fig 1 J). Please add to corresponding legend text the cell type being analyzed.

4. So the reader can more easily interpret and consider the implications of each experiment, consider adding the period of CBZ treatment to the corresponding experiment description in Results.

5. Fig 1A needs statistics

6. Fig 1B - use similar Y axis values as Fig 1A

7. Fig 1K - the image shown is not convincing for the quantitative data, due to poor resolution. For example, the VPA panels look as though red compartments are adjacent to but distinct from green compartments. Showing the green channel as an independent panel may help. Define DIC in the

legend.

8. Fig 2B lacks specificity controls and can be deleted.

9. Fig 2G: The MOI notation is unusual; typically the host cell # is the denominator for MOI, which is one value.

10. Fig 3B. Define hpf

11. Consider distributing the large number of panels over a larger number of separate figures.

Referee #3 (Comments on Novelty/Model System):

The authors clearly demonstrate that carbamazepine a licensed drug has an effect on autophagy in three different model systems, although the mouse data is less emphatic. They have also elegantly dissected a mechanism for one of these drugs. There is a large amount of supporting data of high technical quality, and also using slow growing mycobacteria that are extremely challenging to work with. Although the limited animal data suggests that carbamazepine may not be a drug to take forward into clinical trials it is a very important study because it illustrates the feasibility of host modulation of the autophagosomal pathway as a viable strategy for combating mycobacterial infection.

Referee #3 (Remarks):

The authors have identified two compounds (that are already FDA-approved as anticonvulsants), carbamazepine (CPZ) and valproic acid (VPA), and have demonstrated an antimycobacterial effect in 3 infection models: human macrophages, zebrafish, and mouse. They proceed to demonstrate that this is via induction of autophagy in an mTOR-independent pathway and elegantly elucidate a pathway. In general, the paper is written in clear, accessible language, but with regards to organization and presentation, there are a number of ways in which paper could be improved.

1. The drug screening process, especially given that the paper's title emphasizes this screen is not adequately described. The methods section states that 200 compounds were screened for their ability to kill M. bovis BCG in macrophages, but gives no indication as to what compound library was used, how killing was measured, how many total hits there were, and why CBZ and VPA were chosen going forward. It would be good to know which drugs have been screened and ideally how they compared to CBZ and VPA.

2. It should be pointed out that only CBZ has activity at a clinically applicable concentration

3. Co localization of Rab7 experiment (results illustrated in Figure 1K), was not described at all in materials & methods, and only in a cursory manner in the results section.

4. Figure 1 C - MIC data was presented in a confusing manner. This could be put in the text rather than a figure.

5. Material & methods could do with some rationalization - some of the methods are repeated in the supplementary methods. They would be more helpful to the reader if recorded in the same order that each experiment is addressed in the text, and if more detail was offered in some instances in the paper. Also some detail in the figure legends could be put in the methods.

6. A revision that would significantly improve the clarity of the paper is re-organization of the figures. Some of the information in the figure legends could be moved to the text to reduce bulk in the figures section and improve clarity in the results section. The number of individual panels should be reduced (with some eliminated or moved to supplementary material). The remaining essential panels could be divided into a greater number of figures with each figure representing a separate scientific argument. Within each such figure grouping, the panels could also be re-ordered to better build this argument.

a. For example, here is one possible revision of Figure 1: Identity and dose-dependency screen of compounds that clear intracellular mycobacteria (BCG, H73Rv); demonstrated in mouse macrophages, primary human macrophages, and human alveolar macrophages A, B, D, J (are both d and j required - J is a more comprehensive experiment but shows control of mycobacteria rather than killing this should be stated in the text.

b. Construction of autophagy as the mechanism of action of lead compounds - could even be split into two figures

i. 2-1. Preliminary evidence of autophagy as the mechanism: compounds induce increased autophagosome production E, G, H (in that order)

ii. 2-2. Evidence that the hypothesized process (autophagy) is affected in full (not just the precursor steps of the pathway, ie. autophagosome formation), by these compounds, including measurement of this pathway's outcomes independent of mycobacteria (-synuclein degradation) F, I

iii. 1L: This figure needs a bit more explanation in the text and could be combined with supplementary figure 2. It would also make sense to combine and discuss these results with the immunological experiments presented in figure 4.

c. Figure 2 is also very busy

7. The mice experiments are a bit contradictory. Figure 4a demonstrates an unambiguous effect, although it is one experiment with only one time point showing a difference. However the experiment in 4e shows a much more modest difference for the MDR strain (CSU87). Was this significant? Was this done using the same protocol, if so why has it been presented in such a bizarre way? The experiment should be shown in a conventional manner, using logarithmic decline of CFU as a measure of efficacy. Even if non -significant with CSU87 in the second experiment this still should be presented, and shouldn't impact too negatively on the central message of the paper. The results of the other strains where there is clearly no effect also should be presented. The section on strain differences should be removed altogether and the text altered to say that there was a modest effect seen in mice, only seen against strain CSU87. I would then add that given the prominent effect on lung pathology further experiments need to be done to determine if the modest impact on cfu translates into a survival benefit. A description of the provenance of the strains should be added. It would have been helpful to use well-recognized laboratory strains so that these results could be compared to other work.

8. The histology figures are small. It would be more convincing if there was a quantitative analysis of pulmonary infiltrates presented in the text

9. Ideally I would split the cytokine experiments into a separate figure and combine with those from figure 1 as stated above.

1st Revision - authors' response 11 October 2014

EMM-2014-04137 Response to Reviewers' comments

We thank the reviewers for their enthusiastic comments on the manuscript and summarise our specific responses to their individual points below

Referee #1 (Remarks):

The manuscript from Brown et al. describes a novel and compelling new approach to host-directed TB therapy. The major conclusions of the work are that FDA-approved anti-convulsants (particularly carbamazepine, CBZ) can restrict mycobacterial growth through the induction of autophagy. As this process is mTOR independent, it might be a more specific therapy than immunosuppressants such as rapamycin. The potential of this approach is shown in a variety of infection models, most convincingly in the mouse model.

In general, this is a compelling study and represents a huge amount of work. In fact, it would be easy to imagine that this amount of work would be best presented in multiple manuscripts in which each conclusion were more thoroughly supported. Because so much ground is covered, we are compelled to comment on each conclusion and the experiments required to make each scientifically sound. The major conclusions of the manuscript are:

1. CBZ and VPA induce autophagy in mammalian cells to restrict Mtb. This conclusion is generally well supported. The representation/discussion of these experiments could be improved by:

-Explaining the initial drug screen that led to the identification of CBZ and VPA. No information is provided.

We have now added a detailed description of the initial chemical screening.

-it is unclear how intracellular and extracellular bacteria were differentiated

We accept that the labeling of the graph in Fig 1B is confusing. We have now clarified the figure, figure legend and text accordingly. The left hand axis refers to macrophage-associated viable mycobacteria. Cells were incubated with mycobacteria for 1h, washed and then left for 24h. Supernatants were then removed before cells were lysed and viable mycobacteria enumerated by measuring luminescence. The right hand axis refers to the amounts of viable mycobacteria in the presence of the drugs but without any cells present. We can therefore be confident that the bacteria are extracellular.

-In figure 1C, the "growth/nogrowth" metric is unsatisfying. Actual data would be more customary.

We have utilized broth micro-dilution to evaluate the MICs (as recommended by the CLSI), which defines the MIC as the concentration inhibiting growth (as such it is a growth- no growth metric). We accept that it is best represented as a table and now include this data as Supplementary Table 1).

-scale bars should be added to the images

Scale bars have now been added to all the figures.

-In figure 1I.ii, the ATG12si appears to reduce bacterial numbers to the same degree as CBZ treatment. This suggests that the proper interpretation of the experiment would be that CBZ acts by the inhibition of autophagy, not its activation. This should be addressed or explained.

We would disagree with this interpretation of the data. We often see reduced uptake of bacteria by macrophages in which ATG12 has been knocked down potentially reflected reduced phagocytic capacity. Nevertheless we show that the killing effect of Rapamycin, CBZ and VPA are blocked by knocking down ATG12 to levels that impair the generation of LC3-II.

-Figure 1j should be presented on a log scale, since the bacteria appear to be growing exponentially.
We would disagree. The reason for showing this result on a log scale would be because the data points span a large numerical range. Our data fits nicely on a linear scale and illustrates the significant effects of the compounds on intracellular mycobacteria. Other examples of publications where macrophage-associated CFU are shown on a linear scale include:

Hartman, M. L., & Kornfeld, H. (2011). Interactions between Naive and Infected Macrophages Reduce Mycobacterium tuberculosis Viability. *Plos One*, *6*(11). doi:10.1371/journal.pone.0027972

Fong, C. H. Y., Bebien, M., Didierlaurent, A., Nebauer, R., Hussell, T., Broide, D., … Lawrence, T. (2008). An antiinflammatory role for IKKbeta through the inhibition of "classical" macrophage activation. *The Journal of Experimental Medicine*, *205*(6), 1269–76. doi:10.1084/jem.20080124

Singh, V., Jamwal, S., Jain, R., Verma, P., Gokhale, R., & Rao, K. V. S. (2012). Mycobacterium tuberculosis-driven targeted recalibration of macrophage lipid homeostasis promotes the foamy phenotype. *Cell Host & Microbe*, *12*(5), 669–81. doi:10.1016/j.chom.2012.09.012

Agdestein, A., Jones, A., Flatberg, A., Johansen, T. B., Heffernan, I. A., Djønne, B., … Olsen, I. (2014). Intracellular growth of Mycobacterium avium subspecies and global transcriptional responses in human macrophages after infection. *BMC Genomics*, *15*, 58. doi:10.1186/1471-2164-15-58

-macrophage cell death in response to compound +/- infection should be assessed.
We have added these data to integrate our viability studies on compound alone and have now included it in Supplementary Figure 1.

-Autophagy activation during Mtb infection is not demonstrated. This is important, as the pathways leading to autophagy have been shown to depend on the ESX secretion system that is deleted in BCG. An LC3 I vs II blot would likely suffice.
BCG infection of macrophages has already been shown to induce autophagy (as evidenced by LC3 changes on western blotting). An example of this would be:
Zullo, A. J., & Lee, S. (2012). Mycobacterial induction of autophagy varies by species and occurs independently of mammalian target of rapamycin inhibition. *The Journal of Biological Chemistry*, *287*(16), 12668–78. doi:10.1074/jbc.M111.320135

2. CBZ activation of autophagy is mTOR independent and targets SCN5A
-Throughout figure 2, western blots for SCN5A expression and knockdown would be more convincing and quantitative than immunofluorescence.
We identify SCN5A on primary human macrophages (confirming previous results published by Carrithers, M. D., Dib-Hajj, S., Carrithers, L. M., Tokmoulina, G., Pypaert, M., Jonas, E. a., & Waxman, S. G. (2007). Expression of the Voltage-Gated Sodium Channel NaV1.5 in the Macrophage Late Endosome Regulates Endosomal Acidification. *The Journal of Immunology*, *178*(12), 7822–7832.). The reason for showing the siRNA knockdown is to prove specificity of the antibody. Unfortunately we were unable to get the antibody to work on western blot. Furthermore, the viability of both human cells following siRNA knockdown of SCN5A and bone marrow-derived macrophages from SCN5A+/- mice wert severely compromised, preventing further functional analysis.

-The authors state that, "We first looked for the presence of known molecular targets of CBZ in primary human macrophages and identified the voltage- and stretch- activated sodium channel SCN5A". It would be useful to know what other targets they looked for. Was SCN5A the only one present?

We looked for the presence of a number of voltage-activated channels but could only convincingly identify SCN5A.

-as above, controls for macrophage cell viability during inhibitor treatment would be useful.

We have now added the macrophage viability experiments as requested (Supplementary Figure 5).

-while SLC5A knockdown reduces BCG growth, it is not clear that this knockdown also inhibits autophagy. This should be shown in the context of BCG infection.

We have now included experiments showing that autophagy is increased by SLC5A knockdown. Since addition of mycobacteria to cells will potentially confound autophagy measurements (since there is also an effect of SLC5A on intracellular mycobacterial viability), we have performed these experiments in uninfected cells (Supplementary Figure 6)

3. CBZ induces autophagy by reducing IP3 levels and activating AMPK and ULK

-While the biochemical readouts presented in figure 3H-L are consistent with this model, it seems that these experiments were done in uninfected macrophages. If so, infection could change these results. The model would be strengthened if a subset of these measurements were made in infected cells.

-In Figure 2 Fii, the effect of myo-inositol on BCG growth is confirmed. A simultaneous demonstration that autophagy is altered would strengthen the model.

As mentioned above, measurements of the effects of modulating myo-inositol on autophagy in the presence of mycobacteria will generate confounding results (since the clearance of mycobacteria is also affected by these modifications. We therefore present effects of myo-inositol on autophagy in uninfected macrophages (Figure 4E & Supplementary Figure 7), which are in keeping with previous publications from a number of labs (including our own-);

Sarkar, S., Floto, R. A., Berger, Z., Imarisio, S., Cordenier, A., Pasco, M., … Rubinsztein, D. C. (2005). Lithium induces autophagy by inhibiting inositol monophosphatase. *The Journal of Cell Biology*, *170*(7), 1101–11. doi:10.1083/jcb.200504035

Shaltiel, G., Shamir, A., Shapiro, J., Ding, D. B., Dalton, E., Bialer, M., … Agam, G. (2004). Valproate decreases inositol biosynthesis. *Biological Psychiatry*, *56*(11), 868–874. doi:10.1016/j.biopsych.2004.08.027

Teo, R., King, J., Dalton, E., Ryves, J., Williams, R. S. B., & Harwood, A. J. (2009). PtdIns(3,4,5)P-3 and inositol depletion as a cellular target of mood stabilizers. *Biochemical Society Transactions*, *37*, 1110–1114. doi:10.1042/bst0371110

Criollo, A., Maiuri, M. C., Tasdemir, E., Vitale, I., Fiebig, A. A., Andrews, D., … Kroemer, G. (2007). Regulation of autophagy by the inositol trisphosphate receptor. *Cell Death and Differentiation*, *14*(5), 1029–1039. doi:10.1038/sj.cdd.4402099

Vicencio, J. M., Ortiz, C., Criollo, a, Jones, a W. E., Kepp, O., Galluzzi, L., … Kroemer, G. (2009). The inositol 1,4,5-trisphosphate receptor regulates autophagy through its interaction with Beclin 1. *Cell Death and Differentiation*, *16*(7), 1006–1017. doi:10.1038/cdd.2009.34

-The rationale for using the dictyostelium/M. abscessus model is unclear. It is difficult to believe that this is similar enough to the Mtb/macrophage system for the details of the mechanism to be completely complementary. For these data to be meaningful, the authors would have to show that the ultimate mechanism of bacterial control is the same (i.e. show autophagy is required).
We would disagree with the assertion that dictyostelium does not utilize a complementary system to macrophages to respond to mycobacteria. There are numerous publications utilizing dictyostelium to investigate host restriction of mycobacterial infection including autophagy. We have, however, added more experimental data demonstrating that intracellular killing of mycobacteria is inhibited by autophagy inhibition (Supplementary Figure 8).

Bozzaro, S., & Eichinger, L. (2011). The professional phagocyte Dictyostelium discoideum as a model host for bacterial pathogens. *Current Drug Targets*, *12*(7), 942–54.

Steinert, M., & Heuner, K. (2005). Dictyostelium as host model for pathogenesis. *Cellular Microbiology*, *7*(3), 307–14. doi:10.1111/j.1462-5822.2005.00493.x

Tresse, E., Giusti, C., Kosta, A., Luciani, M.-F., & Golstein, P. (2008). *Autophagy and autophagic cell death in Dictyostelium. Methods in enzymology* (1st ed., Vol. 451, pp. 343–58). Elsevier Inc. doi:10.1016/S0076-6879(08)03223-0

Calvo-garrido, J., Carilla-latorre, S., Kubohara, Y., Santos-rodrigo, N., Mesquita, A., Soldati, T., … Dictyostelium, K. (2010). Genes and pathways , cell death and infection Autophagy in Dictyostelium. *Autophagy*, *6*(6), 686–701.

4. CBZ treatment activates autophagy in zebrafish and controls M. marinum growth.
-These data are a nice complement to the mouse studies. However, more explanation would help. For example, the LC3 images do not appear representative, as the bar graphs show a much smaller increase in autophagosome number than would be predicted by the images presented.
Unfortunately, we found considerable variability of the Carbamazipine and Rapamycin effects between experiments on different days. This may represent problems with solubility of carbamazepine (as has previously been reported (Zakeri-Milani, Barzegar-Jalali, Azimi, & Valizadeh, 2009) (Shayanfar, Velaga, & Jouyban, 2014) and stability of rapamycin (Simamora, Alvarez, & Yalkowsky, 2001)(Jusko & Ferron, 1998)(Il, Alquier, & Maryanoff, 2007). We thought it was fairer to show the averages across experiments performed on different days.

-Do zebrafish have an ortholog for SCN5A?
Zebrafish have a number of presumed voltage-activated Na channels orthologous to SCN5A (including Q20JQ4; A7Y3Y2; Q20JQ5; F1QXF5; Q20JQ8)

-The M. marinum growth during rapamycin treatment is not shown. Why? This would be a great comparison with CBZ.
Unfortunately we have had enormous problems with stability of rapamycin and as a consequence could not dose effectively for the duration required for the infection model.

5. CBZ activates autophagy in vivo in mice. This restricts MDR Mtb growth and enhances immunity.
-These data are generally robust and compelling. However, the presentation of the FACS data could be improved in the following ways:
a. CD11b/CD11a markers are insufficient to differentiate macrophages from DC's. These populations should be referred to by their staining characteristics only.

We have changed the labeling as requested with reference to CD11b and CD11c (rather than CD11a)

b. Gating strategies should be defined.
The gating strategy has been defined in the text as a single cell suspensions of the lungs were incubated with labeled monoclonal antibodies to CD11b, CD11c, to detect macrophages ($CD11b^{high}CD11c^{-}$) and dendritic cells ($CD11b^{-}CD11c^{high}$) by fluorescence-activated cell sorting analysis and referenced (J Infect Dis. (2011) 203 (9): 1240-1248 and J Immunol. 2007 Jul 1;179(1):522-31).

c. Total cell numbers might be more informative than % positive cells. If the authors choose not to present the data this way, the total number of leukocytes (aka. The denominator) should also be provided.
We agree but have removed the LC3+ data comparing infection with different isolates.

d. a brief discussion of the relevance of CM vs EM populations would be helpful.
This has been now added to the main text (page 5).

e. were the lungs profused? This is an important detail to be added to the methods.
Yes, the lungs were perfused and this has been added to the Supplementary Methods section.

5. Different Mtb clinical strains are differentially susceptible to CBZ treatment.
-The LC3 flow cytometry data experiment is interesting, but representative scatter plot should be provided. Also the total cell number (in addition to %) would be useful.
-In panel E the total cfu numbers instead of % reduction should be shown. This is necessary for interpreting panels F and G.
-This is a very interesting observation. The authors rightly state that the mechanism underlying the differential effect of CBZ is unclear. To understand this effect properly, the effect of autophagy would need to be assessed for each strain in cultured macrophages, and additional in vivo work may be required. As a result, the authors should consider removing these data and presenting them in a separate manuscript once the effect is understood.
We agree with the Reviewer. The total CFU numbers are as follows: (SA310: $9.31x10^{7}$; SA310+CBZ: $1.0x10^{8}$; CSU39: $4.14x10^{7}$; CSU39+CBZ: $4.27x10^{7}$).

We have however removed the data for the other TB strains and have added a statement in the text to explain that CBZ had little or no effect on in vivo infection models using two other strains of MTB.

Referee #2 (Comments on Novelty/Model System):
Each of the experimental models applied are well established in the microbial pathogenesis field. The next stage of testing would be non-human primates. However, before extending to that model, I would recommend derivatives of the lead compound be tested to optimize its activity.
We agree with the reviewer and are currently engaged in this project

Referee #2 (Remarks):
In an ambitious study, Brown et al characterize in detail the cellular pathways by which the drug carbamazepine (CPZ) induces autophagy and increases clearance of virulent Mycobacteria from cultured human macrophages, Dictyostelium amoebae, zebrafish, and mice. The authors identify components of the CPZ signaling pathway by applying classical and molecular genetics as well as a logical panel of pharmacological inhibitors in these experimental model systems.
In general, the study is rigorous, quantitative, and thorough. The value of the manuscript is to characterize 1) a non-mTOR-dependent pathway that induces autophagy and 2) a lead compound

that modestly reduces the in vivo burden of some but not all virulent strains of Mycobacterium tuberculosis and that is approved for use in humans as a anticonvulsant. The pressing need for clinical treatments to control MDR and XDR M. tuberculosis raises the value of this cell biological study.
To increase the clarity of the manuscript for a broad readership, several recommendations are provided below.

1. The language of the Abstract and Results should more accurately reflect that the impact of CBZ on pathogen clearance is modest (ie ~ 30% reduction in bacterial burden). For example, line 6 of the Abstract implies a sterilizing clearance of infection by CBZ treatment.
We agree with the reviewer and have changed the text accordingly (page 2 and throughout Discussion.

2. CBZ treatment itself reports to be pro-inflammatory after long treatment periods (yellow lines in Fig 4C). Please discuss.
We have added a statement highlighting this observation (page 5) although we currently do not understand it.

3. Multiple cell types are used as hosts, making it challenging for the reader to interpret panel-to-panel differences (eg Fig 1 A vs Fig 1 Iii vs Fig 1 J). Please add to corresponding legend text the cell type being analyzed.
We have added this information to the legends as requested.

4. So the reader can more easily interpret and consider the implications of each experiment, consider adding the period of CBZ treatment to the corresponding experiment description in Results.
We have added this information to the text as requested.

5. Fig 1A needs statistics
We have added these as requested (Fig 1)

6. Fig 1B - use similar Y-axis values as Fig 1A
We have used the same Y-axis in both these panels as requested

7. Fig 1K - the image shown is not convincing for the quantitative data, due to poor resolution. For example, the VPA panels look as though red compartments are adjacent to but distinct from green compartments. Showing the green channel as an independent panel may help. Define DIC in the legend.
We have removed the Rab7 data from the manuscript for clarity

8. Fig 2B lacks specificity controls and can be deleted.
We have moved Fig 2B to Supplementary Figure 3A. The specificity controls are all shown in Supplementary Figure 3B.

9. Fig 2G: The MOI notation is unusual; typically the host cell # is the denominator for MOI, which is one value.
Figure 2H does not show different MOI but rather different species of PIP_2. We have clarified the text and legends to explain this better.

10. Fig 3B: Define hpf.
We have added a definition of hpf: hours post fertilization.

11. Consider distributing the large number of panels over a larger number of separate figures.
We agree with this suggestion and have spread the data over more figures.

Referee #3 (Comments on Novelty/Model System):
The authors clearly demonstrate that carbamazepine a licensed drug has an effect on autophagy in three different model systems, although the mouse data is less emphatic. They have also elegantly dissected a mechanism for one of these drugs. There is a large amount of supporting data of high technical quality, and also using slow growing mycobacteria that are extremely challenging to work with. Although the limited animal data suggests that carbamazepine may not be a drug to take forward into clinical trials it is a very important study because it illustrates the feasibility of host modulation of the autophagosomal pathway as a viable strategy for combating mycobacterial infection.

Referee #3 (Remarks):
The authors have identified two compounds (that are already FDA-approved as anticonvulsants), carbamazepine (CPZ) and valproic acid (VPA), and have demonstrated an antimycobacterial effect in 3 infection models: human macrophages, zebrafish, and mouse. They proceed to demonstrate that this is via induction of autophagy in an mTOR-independent pathway and elegantly elucidate a pathway. In general, the paper is written in clear, accessible language, but with regards to organization and presentation, there are a number of ways in which paper could be improved.

1. The drug screening process, especially given that the paper's title emphasizes this screen is not adequately described. The methods section states that 200 compounds were screened for their ability to kill M. bovis BCG in macrophages, but gives no indication as to what compound library was used, how killing was measured, how many total hits there were, and why CBZ and VPA were chosen going forward. It would be good to know which drugs have been screened and ideally how they compared to CBZ and VPA.
We have added more information about the screen as requested

2. It should be pointed out that only CBZ has activity at a clinically applicable concentration
We have added a sentence to this effect in the text

3. Co-localization of Rab7 experiment (results illustrated in Figure 1K), was not described at all in materials & methods, and only in a cursory manner in the results section.
We have removed these data from this manuscript and plan to expand these observations in a separate study

4. Figure 1 C - MIC data was presented in a confusing manner. This could be put in the text rather than a figure.
We now describe the MIC values in Supplementary Table 1

5. Material & methods could do with some rationalization - some of the methods are repeated in the supplementary methods. They would be more helpful to the reader if recorded in the same order that each experiment is addressed in the text, and if more detail was offered in some instances in the paper. Also some detail in the figure legends could be put in the methods.
We thank the reviewer for this suggestion and have amended Methods and legends as requested

6. A revision that would significantly improve the clarity of the paper is re-organization of the figures. Some of the information in the figure legends could be moved to the text to reduce bulk in the figures section and improve clarity in the results section. The number of individual panels should be reduced

(with some eliminated or moved to supplementary material). The remaining essential panels could be divided into a greater number of figures with each figure representing a separate scientific argument. Within each such figure grouping, the panels could also be re-ordered to better build this argument:

a. For example, here is one possible revision of Figure 1: Identity and dose-dependency screen of compounds that clear intracellular mycobacteria (BCG, H73Rv); demonstrated in mouse macrophages, primary human macrophages, and human alveolar macrophages A, B, D, J (are both d and j required - J is a more comprehensive experiment but shows control of mycobacteria rather than killing this should be stated in the text.

b. Construction of autophagy as the mechanism of action of lead compounds - could even be split into two figures
i. 2-1. Preliminary evidence of autophagy as the mechanism: compounds induce increased autophagosome production E, G, H (in that order)
ii. 2-2. Evidence that the hypothesized process (autophagy) is affected in full (not just the precursor steps of the pathway, ie. autophagosome formation), by these compounds, including measurement of this pathway's outcomes independent of mycobacteria (α-synuclein degradation) F, I
iii. 1L: This figure needs a bit more explanation in the text and could be combined with supplementary figure 2. It would also make sense to combine and discuss these results with the immunological experiments presented in Figure 4.
c. Figure 2 is also very busy
We have changed this as requested.

7. The mice experiments are a bit contradictory. Figure 4a demonstrates an unambiguous effect, although it is one experiment with only one time point showing a difference. However the experiment in 4e shows a much more modest difference for the MDR strain (CSU87). Was this significant? Was this done using the same protocol, if so why has it been presented in such a bizarre way? The experiment should be shown in a conventional manner, using logarithmic decline of CFU as a measure of efficacy. Even if non -significant with CSU87 in the second experiment this still should be presented, and shouldn't impact too negatively on the central message of the paper. The results of the other strains where there is clearly no effect also should be presented. The section on strain differences should be removed altogether and the text altered to say that there was a modest effect seen in mice, only seen against strain CSU87. I would then add that given the prominent effect on lung pathology, further experiments need to be done to determine if the modest impact on cfu translates into a survival benefit. A description of the provenance of the strains should be added. It would have been helpful to use well-recognized laboratory strains so that these results could be compared to other work.
We have replaced the bacterial growth figures using logarithmic decline of CFU as a measure of efficacy. The bacterial strains used have been characterized in previous publication, which we have added to the text: CSU87 (Tuberculosis (Edinb). 2014 94:140-7; J Leukoc Biol. 2006, 79:80-6); CSU SA310 (Tuberculosis (Edinb). 2009, 89:203–9); CSU39 (Infect. Immun. 1997, 65:1189).

8. The histology figures are small. It would be more convincing if there was a quantitative analysis of pulmonary infiltrates presented in the text
We have now included lesion scores for the pulmonary infiltrates presented in the text.

9. Ideally I would split the cytokine experiments into a separate figure and combine with those from figure 1, as stated above.
We have now modified the figures as requested.

2nd Editorial Decision 03 November 2014

Thank you for the submission of your revised manuscript to EMBO Molecular Medicine. We have now received the enclosed report from the referee asked to re-assess it. As you will see this reviewer is now supportive and I am pleased to inform you that we will be able to accept your manuscript pending editorial final amendments.

Please submit your revised manuscript within two weeks. I look forward to seeing a revised form of your manuscript as soon as possible.

***** Reviewer's comments *****

Referee #1 (Remarks):

The authors have remedied essentially all of my concerns. Only one issue remains. In the initial review, I indicated that it is "important" to show that at least the major in vitro observations could be recapitulated using M. tuberculosis instead of BCG. This is because a major STING-dependent selective autophagy pathway is ony triggered by bacteria expressing an intact ESX secretion system. This system is deleted in BCG. A major point of this manuscript is that multiple pathways can lead to autophagy. As a result, the author's simple argument (that BCG triggers autophagy and therefore their model is appropriate) is not really sufficient. How do they know that the same pathways will be relevant when M. tuberculosis is used and the STING pathway predominates?

In the end, this is a relatively minor point, since the authors have shown effects in M. tuberculosis-infected mice. However, it will remain difficult to integrate these observations with the rest of the field unless in vitro data with M. tuberculosis is provided.
